# The Challenge of Stratifying Obesity: Attempts in the Quebec Family Study

**DOI:** 10.3389/fgene.2019.00994

**Published:** 2019-10-10

**Authors:** Juan de Toro-Martín, Frédéric Guénard, Claude Bouchard, Angelo Tremblay, Louis Pérusse, Marie-Claude Vohl

**Affiliations:** ^1^Institute of Nutrition and Functional Foods (INAF), Université Laval, Quebec, QC, Canada; ^2^School of Nutrition, Université Laval, Quebec, QC, Canada; ^3^Human Genomics Laboratory, Pennington Biomedical Research Center, Baton Rouge, LA, United States; ^4^Department of Kinesiology, Université Laval, Quebec, QC, Canada; ^5^Quebec Heart and Lung Institute Research Center, Quebec, QC, Canada

**Keywords:** polygenic risk score, obesity, genetics, genome-wide association study, body mass index

## Abstract

**Background and aims:** Obesity is a major health problem worldwide. Given the heterogeneous obesity phenotype, an optimal obesity stratification would improve clinical management. Since obesity has a strong genetic component, we aimed to develop a polygenic risk score (PRS) to stratify obesity according to the genetic background of the individuals.

**Methods:** A total of 231 single nucleotide polymorphisms (SNP) significantly associated to body mass index (BMI) from 21 genome-wide association studies were genotyped or imputed in 881 subjects from the Quebec Family Study (QFS). The population was randomly split into discovery (80%; n = 704) and validation (20%; n = 177) samples with similar obesity (BMI ≥ 30) prevalence (27.8% and 28.2%, respectively). Family-based associations with obesity were tested for every SNP in the discovery sample and a weighed and continuous PRS_231_ was constructed. Generalized linear mixed effects models were used to test the association of PRS_231_ with obesity in the QFS discovery sample and validated in the QFS replication sample. Furthermore, the Fatty Acid Sensor (FAS) Study (n = 141; 27.7% obesity prevalence) was used as an independent sample to replicate the results.

**Results:** The linear trend test demonstrated a significant association of PRS_231_ with obesity in the QFS discovery sample (OR_trend_ = 1.19 [95% CI, 1.14-1.24]; P = 2.0x10^-16^). We also found that the obesity prevalence was significantly greater in the higher PRS_231_ quintiles compared to the lowest quintile. Significant and consistent results were obtained in the QFS validation sample for both the linear trend test (OR_trend_ = 1.16 [95% CI, 1.07-1.26]; P = 6.7x10^-4^), and obesity prevalence across quintiles. These results were partially replicated in the FAS sample (OR_trend_ = 1.12 [95% CI, 1.02-1.24]; P = 2.2x10^-2^). PRS_231_ explained 7.5%, 3.2%, and 1.2% of BMI variance in QFS discovery, QFS validation, and FAS samples, respectively.

**Conclusions:** These results revealed that genetic background in the form of a 231 BMI-associated PRS has a significant impact on obesity, but a limited potential to accurately stratify it. Further studies are encouraged on larger populations.

## Introduction

Obesity is a metabolic condition characterized by a large heterogeneity. Body mass index (BMI) has been widely used as a reference indicator to characterize the different degrees of obesity (Seidell and Flegal, 1997). Although other indicators, such as body fat percentage or waist-to-hip ratio, have been also employed (Ashwell et al., 1985; Lean et al., 1995), BMI remains the most commonly used in clinical practice. Different approaches have been explored to stratify obesity based on BMI classification (Li et al., 2010a; Peterson et al., 2011). Among others, an obesity background during childhood and a familial history of obesity remain as the lead traditional risk factors of obesity (Loos and Janssens, 2017). In this regard, the Quebec Family Study (QFS) has focused on traditional and nontraditional risk factors of obesity, adiposity, or body fat distribution and their genetic determinants (Chaput et al., 2014), leading to a body of evidence on the genetic and familial environmental background for the development of obesity (Robitaille et al., 2003; Do et al., 2008; Choquette et al., 2012).

Obesity is phenotypically and genetically highly complex (Ghosh and Bouchard, 2017) and, in spite of the growing evidence linking genetics to obesity, the use of genetic information to correctly classify obesity has led to heterogeneous results. Although many genetic variants have been repeatedly associated with obesity, such as those located within *FTO* or *MC4R* genes (Frayling et al., 2007; Loos et al., 2008; Rouskas et al., 2012), their ability to stratify obesity remains insufficient, as compared to traditional risk factors or familial resemblance (Loos and Janssens, 2017). In an effort to overcome this limitation, the use of a combination of obesity-associated SNPs has become a promising strategy. A number of studies have already tested the ability to stratify obesity based on a cumulative number of BMI-associated SNPs combined into a single parameter, commonly called polygenic risk score (PRS) (Li et al., 2010a; Sandholt et al., 2010; Speliotes et al., 2010; Peterson et al., 2011; Hung et al., 2015; Locke et al., 2015). By using this approach, obesity stratification results from a combination of SNPs, which offers a more integrated view of the genetic basis of obesity.

To expand our knowledge on the genetic basis of obesity, the ability of a PRS constructed with all the obesity-associated SNPs currently known was tested. The QFS, a study designed to investigate the genetic and environmental factors of obesity, was used to construct and test the PRS.

## Material and Methods

### Population

Cross-sectional data from 881 QFS participants were used in the present study. The QFS is a study conducted from 1979 to 2002 in several phases, which focuses the genetic determinants of obesity and body fat distribution (Chaput et al., 2014). The 881 participants of the present study were from 222 French-Canadian nuclear families from Quebec City and were recruited according to their obesity status (at least one parent and one offspring with a BMI of 32 kg/m^2^ or higher) (Chaput et al., 2014). The participants were randomly split into the QFS discovery (80%; 704 participants) and QFS validation (20%; 177 participants) samples.

The replication sample comprised 141 subjects from the Fatty Acid Sensor (FAS) Study, in which subjects from the Quebec City metropolitan area were originally recruited to identify determinants of the plasma triglyceride response to an n-3 fatty acid supplementation (registered at ClinicalTrials.gov as NCT01343342). Trial details and participant selection criteria are extensively described in (Rudkowska et al., 2014). Briefly, participants of the FAS study were metabolically healthy subjects with a BMI between 25 and 40 kg/m^2^ and not taking any medication to treat lipid disorders or fatty acid supplements for at least 6 months prior to the intervention. From a total of 254 subjects included in the study, 210 completed the intervention protocol, and those 141 exhibiting the most extreme triglyceride response after the supplementation were selected to perform a genome wide association study (GWAS).

Experimental protocols of both QFS and FAS studies were approved by the ethic committee of the Laval University and were conducted in accordance with the Declaration of Helsinki. Participants of both studies provided written informed consent.

### Genotyping and Imputing

A total of 231 single nucleotide polymorphisms (SNPs) previously associated with BMI were selected from the NHGRI-EBI GWAS catalog (33) by using “body mass index” as both search term and disease/trait filter on November 2015 (**Table S1**). Concretely, 163 SNPs significantly associated (P < 5×10^−8^) with BMI and 68 SNPs showing suggestive evidence of association (P < 1×10^−6^) were selected from 16 previous GWAS and 6 GWAS meta-analysis. From the 231 selected SNPs, 96 were previously genotyped in the QFS sample using the Illumina 610-Quad chip containing 620,901 markers, as described in detail elsewhere (Sung et al., 2016). Imputation of remaining 135 SNPs was performed using MaCH software (Li et al., 2010b) and the CEU reference panel consisting of 120 haplotypes from HapMap Phase II data (release 22, build 36), as previously described (Sung et al., 2016).

The 141 participants from the FAS study were genotyped using the Illumina HumanOmni-5-Quad Bead-Chip (Illumina, San Diego, CA), containing 4,301,331 markers, from which 153 belonged to the 231 selected BMI-associated SNPs in the present study. The mean call rate across all samples was 99.84%. None of the 141 samples analyzed were excluded due to low signal intensity or low overall call rate (<95%). Inclusion criteria for BMI-associated SNPs were MAF > 1%, call rate > 95%, and HWE P > 2.6x10^-4^. Imputation of the remaining 78 SNPs was performed in the Michigan Imputation Server using the 1000G Phase 3 (Version 5) as reference panel and Minimac3 as imputation software (Das et al., 2016). The mean imputation rate was 0.93 and none of the 78 imputed SNPs were excluded due to low imputation quality (r^2^ > 0.3) (Li et al., 2010b).

Sample quality, call rate, allele frequencies, and HWE tests were assessed using PLINK 1.9 (Chang et al., 2015). Linkage disequilibrium (LD) was not considered as an SNP filtering strategy in the present study in order to maximize the number of available SNPs for PRS construction (Chatterjee et al., 2013; Khera et al., 2018). None of the SNPs were excluded based on HWE and MAF criteria, leaving the 231 directly genotyped or imputed BMI-associated SNPs for statistical analyses (**Table S1**). All of the 231 SNPs had a MAF > 1% in each the QFS discovery, QFS validation, and FAS replication samples.

### Polygenic Risk Score

**Figure 1:**
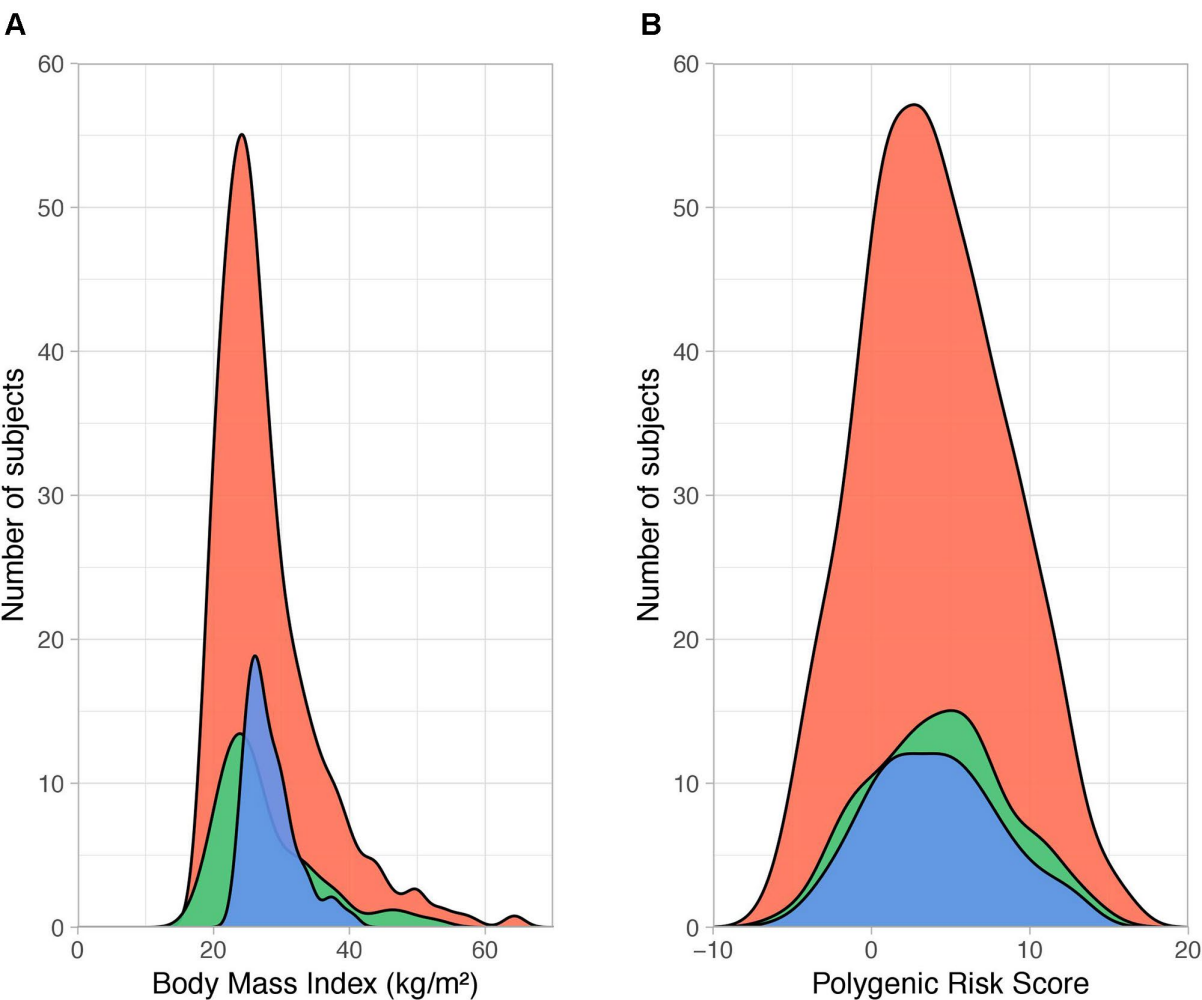
Distribution of body mass index and polygenic risk score PRS_231_. **(A)** Density plot showing the distribution of body mass index (BMI, calculated as weight in kilograms divided by the square of height in meters, kg/m^2^) across QFS discovery (red), QFS validation (green) and FAS replication (blue) samples. The dispersion of BMI data was larger in both the QFS discovery (SD = 7.62) and QFS validation samples (SD = 7.46) than in the FAS replication sample (SD = 3.76). **(B)** Density distribution of polygenic risk score PRS_231_ values across all participants (with and without obesity) in the three study samples. Red, green and blue colors stand for QFS discovery, QFS validation and FAS replication samples, respectively. QFS and FAS stand for Quebec Family Study and Fatty Acid Sensor Study, respectively.

Family-based case-control associations with obesity defined as BMI ≥ 30 kg/m^2^ were tested for every SNP in the QFS discovery sample taking into account familiar relationships. An additive model of inheritance was used to test genetic associations. Results from single-SNP association tests with obesity status led to the construction of a weighted PRS by summing the number of alleles of all the 231 BMI-associated SNPs (PRS_231_) multiplied by their odds ratios (OR). The sum of weighed alleles resulted in a continuous score (**Figure 1**), whose ability to stratify obesity was subsequently tested. First, the power of PRS_231_ to stratify obesity was evaluated in the QFS discovery sample, and its utility was validated in the QFS validation sample. The ability of PRS_231_ was independently tested in the FAS replication sample. Furthermore, PRS_231_ was categorized into quintiles to examine its association with the prevalence of obesity. Finally, the proportion of BMI variance explained by PRS_231_ was quantified. All the analyses were carried out in QFS and replicated in the independent FAS study.

### Statistical Analysis

Single-SNP association analyses with obesity were performed using a generalized linear mixed effects model integrating family data (Chen and Yang, 2010). A binary obesity variable (BMI > 30 kg/m^2^) was used as outcome, and sex and age were included as covariates in the model. Furthermore, the association of PRS_231_ with obesity was evaluated by means of generalized linear (binomial with logit link function) and linear mixed models with flexible covariance structure to account for family relatedness (Ziyatdinov et al., 2018). All statistical procedures were carried out in R version 3.5.0 (R Core Team, 2018) (https://www.R-project.org). GWAF package (Chen and Yang, 2010) was used to test single-SNP association analyses, and lme4qtl (Ziyatdinov et al., 2018) to test PRS_231_ association with obesity in QFS, given their ability to take into account family relatedness. Hmisc package was used to evaluated the performance of classification models (Harrell, 2018).

#### Generalized Linear Mixed Model

The full generalized linear mixed model included obesity as a binary outcome and sex, age, and PRS_231_ as fixed effects. Family relatedness was included as random effect in the form of a kinship matrix. Results from the linear trend test (OR_trend_) were used to analyze the association of the continuous PRS_231_ with obesity. The association of PRS_231_ with obesity was also tested among PRS_231_ quintiles.

#### Evaluation of PRS_231_ Performance

Using the predicted probability of obesity from generalized linear mixed models, the association of PRS_231_ with obesity was tested as the area under a receiver operating characteristic curve (AUC_ROC_). Results were adjusted for optimism (AUC_adj_) by bootstrapping (n = 1000), obtaining bias-corrected 95% confidence intervals of the difference (95%CI_diff_) for further AUC_adj_ comparison, ultimately used to determine the overall performance of the different models. The added value of different models was tested by calculating the differences between bootstrapped AUC_adj_, which was considered significant when 95%CI_diff_ did not contain zero. Two alternative methods to AUC_ROC_ of assessing improvement in model performance, the net reclassification index (NRI), and the integrated discrimination index (IDI) (Pencina et al., 2008) were also used to evaluate the net effect accomplished by adding a PRS to the model.

#### Linear Mixed Model

A linear mixed model with flexible covariance structure to account for family relatedness was fit to test the association between PRS_231_ and BMI. The full linear mixed model included BMI as a quantitative outcome and sex, age, and PRS_231_ as fixed effects. Family relatedness was included as random effect. Finally, results from the linear mixed model were used to calculate the proportion of BMI variance explained by PRS_231_ within each sample.

#### Power Calculations

Power calculations were performed using the package AVENGEME (Dudbridge, 2013; Palla and Dudbridge, 2015), which is able to calculate the power of a PRS, derived from a training sample, to correctly classify traits in a target sample. Herein, the QFS discovery sample was used as training sample, whereas QFS validation and FAS replication samples were used as target samples. Statistical power was then calculated based on the obesity prevalence in each sample, on the number of SNPs in the polygenic score, and on the proportion of variance explained by the polygenic score in the training sample. A significance level of 0.05 for association testing between the polygenic score and obesity prevalence in target samples was also used to calculate statistical power.

## Results

### Patients

The main clinical characteristics of QFS and FAS participants are depicted in **Table 1**. The QFS cohort (n = 881) was randomly split into discovery (80%; n = 704) and validation (20%; n = 177) samples with similar obesity (BMI ≥ 30kg/m^2^) prevalence (27.8% and 28.2%, respectively). The replication sample was composed of participants from the FAS study (n = 144; 27.7% obesity prevalence). The distribution of BMI data within each sample was significantly different (Bartlett P value = 2.2x10^-16^). As shown in **Figure 1**, the dispersion of BMI data was larger in both QFS discovery (SD = 7.62) and QFS validation samples (SD = 7.46) than in the FAS replication sample (SD = 3.76). As shown in **Table 1**, higher proportions of women with obesity were found in both QFS discovery and FAS replication samples, but not in the QFS validation sample. No significant differences in age or height were found between participants with and without obesity among all samples (**Table 1**).

**Table 1:** Clinical characteristics of subjects.

	QFS Discovery		P value	QFS Validation		P value	FAS Replication		P value
	Control	Obesity		Control	Obesity		Control	Obesity	
Sex (male/female)	235/273	68/128	0.01	50/77	27/23	0.08	55/47	13/26	0.03
Age (years)	42.0 ± 17.4	44.7 ± 15.7	0.06	41.8 ± 16.3	46.1 ± 13.2	0.09	30.9 ± 8.5	33.4 ± 9.6	0.14
Height (m)	1.7 ± 0.1	1.6 ± 0.1	0.08	1.6 ± 0.1	1.7 ± 0.1	0.19	1.7 ± 0.1	1.7 ± 0.1	0.28
Weight (kg)	66.6 ± 11.0	101.7 ± 20.7	<0.0001	65.0 ± 11.3	104.7 ± 19.4	<0.0001	78.5 ± 10.3	96.5 ± 14.7	<0.0001
BMI (kg/m^2^)	24.1 ± 3.0	37.8 ± 7.2	<0.0001	23.8 ± 3.0	37.5 ± 6.3	<0.0001	26.5 ± 1.7	33.4 ± 3.1	<0.0001
Waist circumference (cm)	81.1 ± 10.7	111.0 ± 15.1	<0.0001	78.9 ± 10.2	113.8 ± 12.6	<0.0001	85.6 ± 6.0	88.3 ± 7.8	0.03
Triglycerides (mmol/l)	1.45 ± 1.59	1.92 ± 0.85	<0.0001	1.26 ± 0.62	2.18 ± 1.50	<0.0001	1.23 ± 0.66	1.55 ± 0.70	0.01
Total cholesterol (mmol/l)	5.00 ± 1.18	5.00 ± 1.03	0.99	4.92 ± 0.91	4.91 ± 0.90	0.95	4.79 ± 0.94	5.18 ± 0.99	0.03
HDL cholesterol (mmol/l)	1.27 ± 0.31	1.09 ± 0.27	<0.0001	1.39 ± 0.36	0.97 ± 0.21	<0.0001	1.42 ± 0.35	1.41 ± 0.46	0.89
LDL cholesterol (mmol/l)	3.09 ± 0.93	3.05 ± 0.90	0.61	2.98 ± 0.76	3.01 ± 0.76	0.81	2.80 ± 0.88	3.07 ± 0.87	0.10
SBP (mm Hg)	118.6 ± 19.6	126.6 ± 20.5	<0.0001	114.9 ± 18.2	127.1 ± 15.7	<0.0001	112.7 ± 12.4	113.7 ± 12.1	0.65
DBP (mm Hg)	71.8 ± 9.7	76.6 ± 10.7	<0.0001	69.7 ± 9.6	77.6 ± 9.8	<0.0001	67.0 ± 8.4	72.5 ± 7.3	0.0004
Fasting glucose (mmol/l)	5.18 ± 0.96	5.78 ± 1.64	<0.0001	5.06 ± 0.79	6.23 ± 2.30	<0.0001	4.91 ± 0.44	5.23 ± 0.42	0.0001
Fasting insulin (mU/l)	5.67 ± 3.51	12.77 ± 7.60	<0.0001	5.10 ± 3.43	12.99 ± 8.37	<0.0001	7.61 ± 3.17	10.51 ± 4.43	<0.0001
HOMA-IR	1.37 ± 1.19	3.44 ± 2.85	<0.0001	1.17 ± 0.87	3.65 ± 2.93	<0.0001	1.66 ± 0.66	2.43 ± 1.01	<0.0001

Data are presented as means ± SD or number of participants (sex). Male/female ratio was analyzed by the χ^2^-test. Quantitative traits were analyzed using the independent t-test. BMI, body mass index; HDL, high-density lipoprotein; LDL, low-density lipoprotein; SBP, systolic blood pressure; DBP, diastolic blood pressure; HOMA-IR, homeostatic model assessment of insulin resistance (calculated as fasting glucose (mmol/l) x Fasting insulin (mU/ml) / 22.5).

### Impact of PRS_231_ on Obesity Prevalence

**Figure 2:**
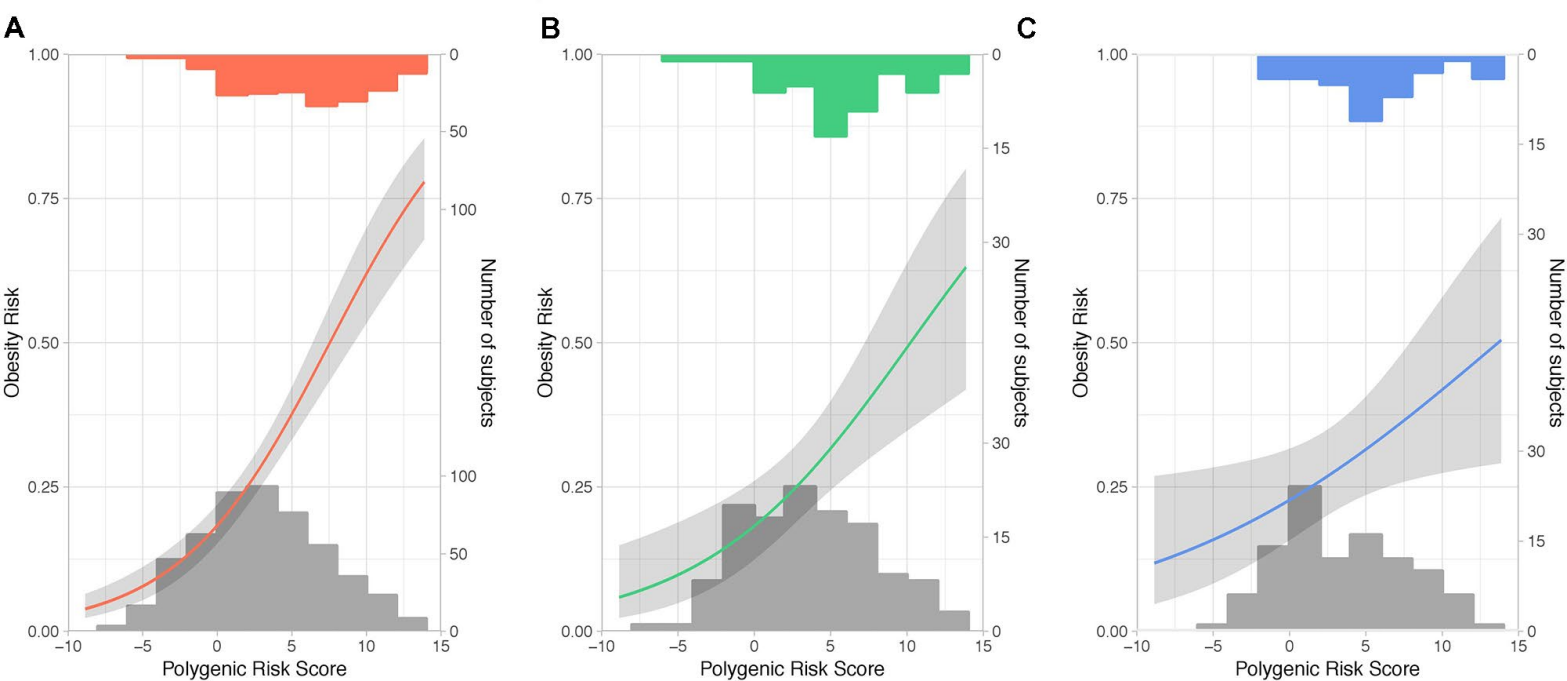
Polygenic risk score PRS_231_ was significantly associated with obesity. Fitted generalized linear mixed models with 95% confidence intervals (grey shade) for the likelihood of obesity, defined as a binary variable (body mass index ≥ 30kg/m^2^), by polygenic risk score PRS_231_. The final model included age, sex and polygenic risk score PRS_231_ as fixed effects, and family relatedness as random effect. Predicted probabilities of obesity between 0 and 1 are related to fixed effects and conditioned on random effect. From left to right: **(A)** QFS discovery, **(B)** QFS validation, and **(C)** FAS replication samples. Grey bars at the bottom of plots represent the distribution of PRS_231_ across subjects without obesity. Red, green and blue bars at the top of plots represent the distribution of PRS_231_ across subjects with obesity in the **(A)** QFS discovery, **(B)** QFS validation, and **(C)** FAS replication samples, respectively. QFS and FAS stand for Quebec Family Study and Fatty Acid Sensor Study, respectively.

Single-SNP association tests based on generalized linear mixed models revealed that only 31 of 231 SNPs (**Table S1**) showed a significant association (P < 0.05) with obesity (**Table S2**). Among 11 SNPs showing a higher frequency of rare alleles in participants without obesity, 7 mapped to the *TMEM18* locus. On the other hand, for 20 SNPs the rare allele was significantly more prevalent in the group having a BMI greater than 30 kg/m^2^, with *FTO* being the most common locus. The statistical power to detect significant associations between the polygenic score and obesity prevalence was 0.79 in both QFS validation and FAS replication samples when using all the 231 SNPs, whereas it decreased to 0.52 when using only the 31 significant SNPs. In view of that, all the 231 SNPs were further used to build a weighed and continuous PRS (PRS_231_) to assess its association with obesity prevalence. We first tested the impact of PRS_231_ in the QFS discovery sample and results demonstrated a highly significant association with obesity (OR_trend_ = 1.24, 95%_CI_ = 1.17-1.31, P = 1.03x10^-13^). As shown in **Figure 2A**, participants with obesity had higher PRS_231_ values than those without obesity. Likewise, significant and consistent results were obtained for the linear trend test in the QFS validation sample (OR_trend_ = 1.19, 95%_CI_ = 1.06-1.33, P = 4.1x10^-3^) (**Figure 2B**). Finally, the significant association between PRS_231_ and obesity was independently replicated in the FAS sample (OR_trend_ = 1.12, 95%_CI_ = 1.01-1.25, P = 3.6x10^-2^) (**Figure 2C**).

### PRS_231_ and Obesity Prevalence

**Figure 3:**
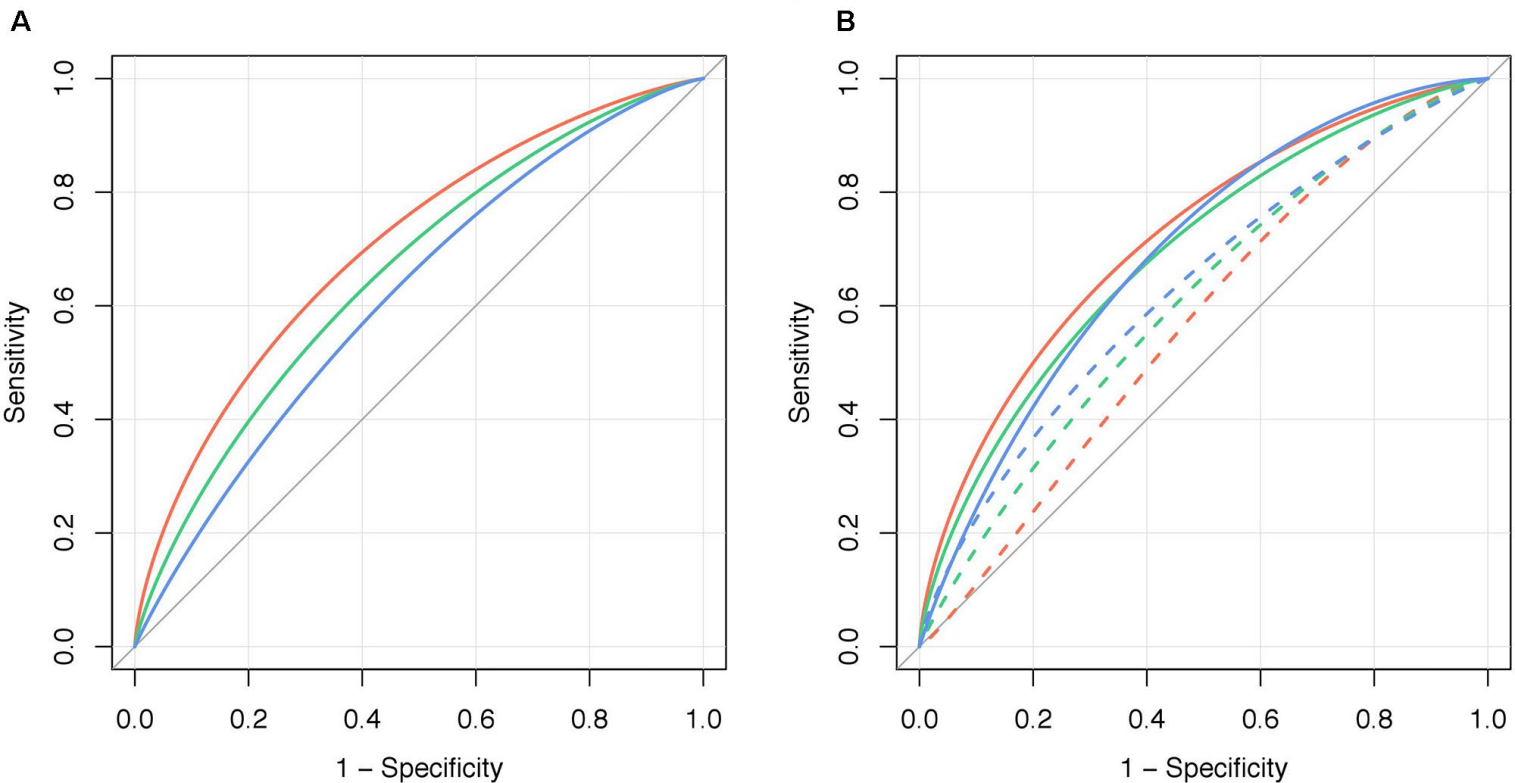
Polygenic risk score PRS_231_ significantly increased the ability to stratify obesity. **(A)** Graphical representation of the area under the ROC curve for obesity, defined as a binary variable (body mass index ≥ 30kg/m^2^), adjusted for bootstrapping (AUC_adj_, n=1000), for polygenic risk score PRS_231_ alone (“PRS_231_”) in the QFS discovery (red line), QFS validation (green line) and FAS replication (blue line) samples. **(B)** The increase in the ability to stratify obesity by PRS_231_ was calculated as the difference between AUC_adj_ of the final models including sex and age as fixed effects before (“Model,” dashed lines) and after adding polygenic risk score PRS_231_ (“Model+PRS_231_”, solid lines). QFS and FAS stand for Quebec Family Study and Fatty Acid Sensor Study, respectively.

The ability of PRS_231_ to associate with obesity *per se* was higher in the QFS discovery sample (AUC_adj_ = 0.704) than that observed in the QFS validation sample (AUC_adj_ = 0.661) and in the FAS replication sample (AUC_adj_ = 0.619) (**Figure 3A**). The addition of PRS_231_ into the full model including sex and age as fixed effects provided a significant increase in overall classification accuracy in both the QFS discovery (AUC_adj_ = 0.141, 95%_CIdiff_ = 0.09-0.19) and QFS replication samples (AUC_adj_ = 0.127, 95%_CIdiff_ = 0.04-0.22), but not in the FAS replication sample (AUC_adj_ = 0.060, 95%_CIdiff_ = -0.01-0.13) (**Figure 3B**). After the inclusion of PRS_231_ into the model, a significant improvement in the correct classification of individuals with obesity was found in the QFS discovery sample for both NRI (0.634; 95%CI = 0.477-0.791; P = 2.7x10^-15^) and IDI (0.129; 95%CI = 0.098-0.159; P = 3.4x10^-17^). Similar results were found in the QFS validation sample for both NRI (0.346; 95%CI = 0.048-0.644; P = 2.3x10^-2^) and IDI (0.069; 95%CI = 0.024-0.114; P = 2.9x10^-3^). Although the inclusion of PRS_231_ did not increase the accuracy of the model in the FAS replication sample, the reclassification analysis also showed that both NRI (0.376; 95%CI = 0.013-0.738; P = 4.2x10^-2^) and IDI (0.035; 95%CI = 0.002-0.069; P = 3.8x10^-2^) significantly increased after adding PRS_231_.

### Impact of PRS_231_ Quintiles on the Obesity Prevalence

**Figure 4:**
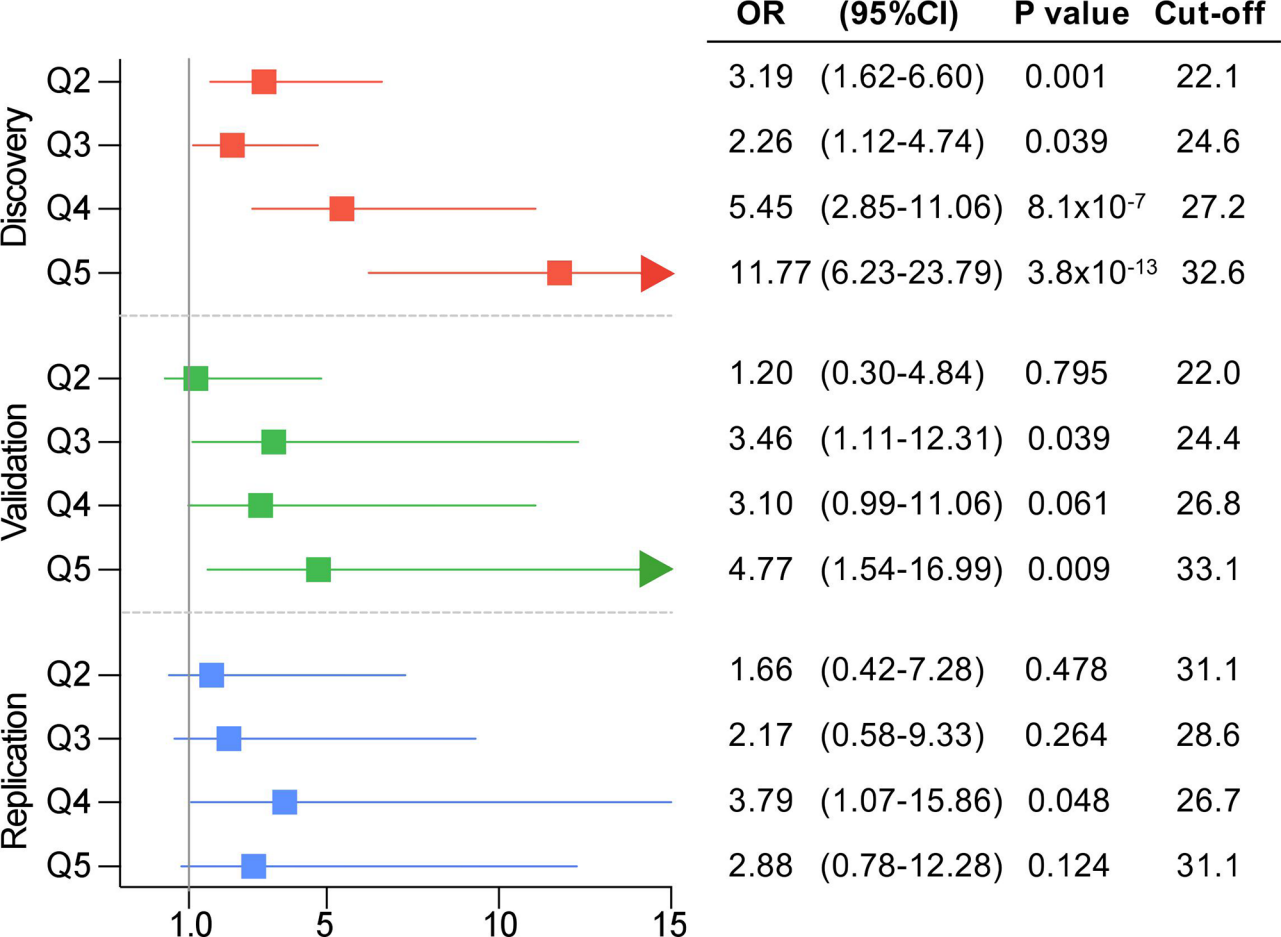
Obesity prevalence consistently increased across polygenic risk score PRS_231_ quintiles. Forest plot for obesity prevalence, defined as a binary variable (body mass index ≥ 30kg/m^2^), across quintiles in the three study samples. From up to bottom: QFS discovery (red), QFS validation (green) and FAS replication (green) samples. Odds ratio (OR) and 95% confidence intervals (95%CI) are calculated for quintiles Q2, Q3, Q4, and Q5 compared to the lowest quintile Q1. Cut-off stands for body mass index (BMI) cut-off points of each quintile. P values were obtained by means of generalized linear mixed models (binomial with logit link function) models including age, sex, and PRS_231_ quintiles as fixed effects and family relatedness as random effect, with flexible covariance structure to account for family relatedness. QFS and FAS stand for Quebec Family Study and Fatty Acid Sensor Study, respectively.

In order to stratify obesity according to genetic background, patients were categorized into PRS_231_ quintiles. Again, results showed that obesity prevalence was significantly higher among upper PRS_231_ quintiles, as compared to the lowest quintile, in the QFS discovery sample (**Figure 4**). In agreement with these results, participants in the third and fifth quintiles in the QFS validation sample also showed a significantly increased prevalence of obesity, as compared to the lowest quintile (**Figure 4**). Lastly, although participants from all quintiles in the FAS replication sample showed greater obesity prevalence, only those into the fourth quintile showed a significant increase (**Figure 4**).

### Quantitative Impact of PRS_231_ on BMI

**Figure 5:**
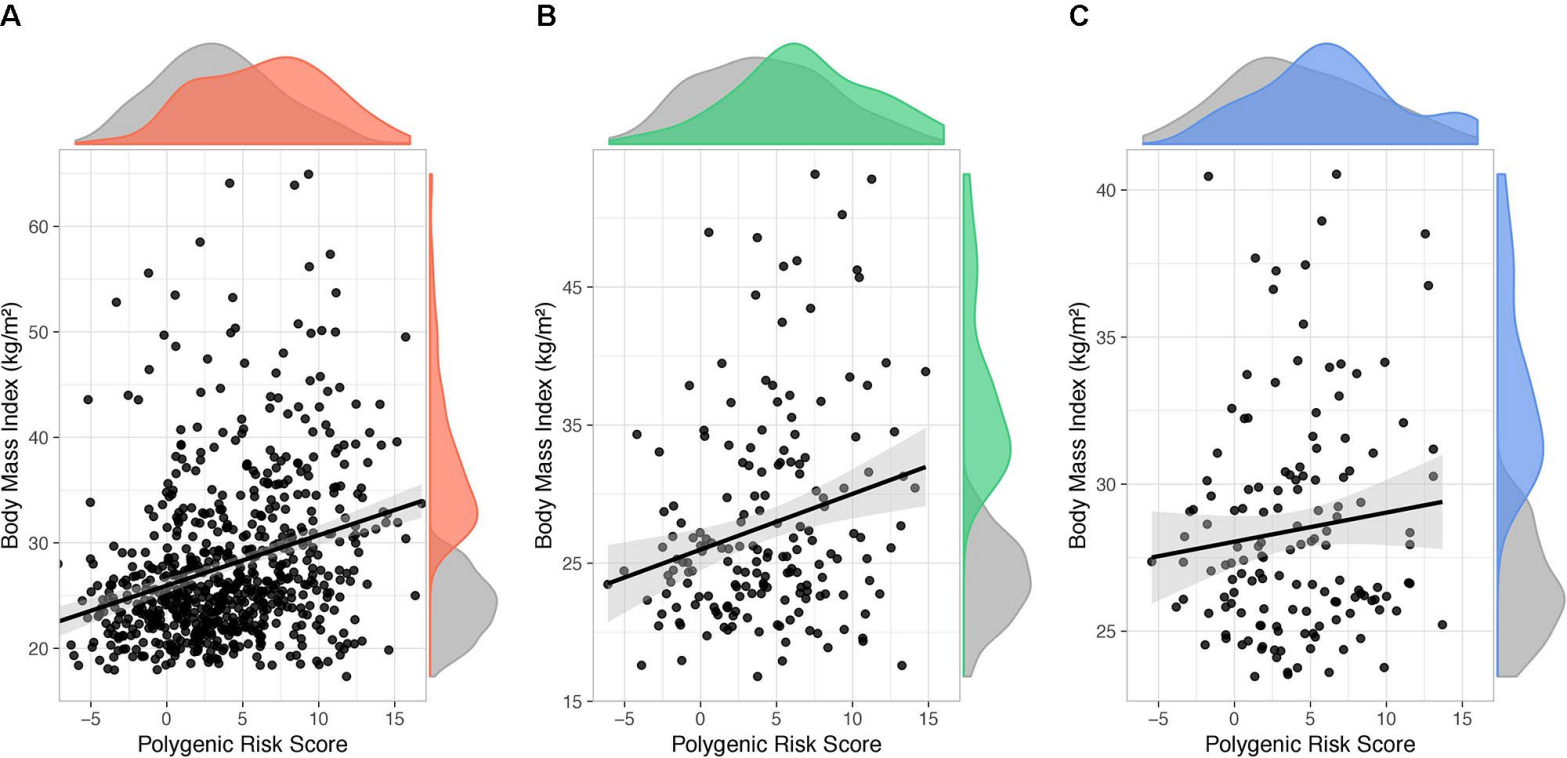
Polygenic risk score PRS_231_ had a significant impact on body mass index. Scatter plots showing fitted linear mixed models including age, sex, and polygenic risk score PRS_231_ as fixed effects and family relatedness as random effect, with flexible covariance structure to account for family relatedness. The association between PRS_231_ and body mass index (BMI), measured as a continuous variable, with 95% confidence intervals (grey shade) is shown in **(A)** QFS discovery, **(B)** QFS validation, and **(C)** FAS replication samples. Density distributions of PRS_231_ and BMI are shown at the top and right of plots, respectively. Grey color represents subjects without obesity. Red, green and blue colors represent subjects with obesity in the **(A)** QFS discovery, **(B)** QFS validation, and **(C)** FAS replication samples, respectively. QFS and FAS stand for Quebec Family Study and Fatty Acid Sensor Study, respectively.

In order to estimate the BMI variance accounted for by PRS_231_, a linear mixed model with BMI as quantitative outcome and sex and age as covariates was used. With obesity defined as a binary outcome, significant effect of PRS_231_ on BMI was observed in the QFS discovery sample (β = 0.46; P = 6.9x10^-13^) (**Figure 5A**). A significant effect was also reported in the QFS replication sample, but to a lesser extent (β = 0.33; P = 1.2x10^-2^) (**Figure 5B**). Finally, the effect of PRS_231_ on BMI in the FAS replication sample was directionally consistent with that observed in both QFS samples, although not statistically significant, (β = 0.10; P = 0.18) (**Figure 5C**). PRS_231_ explained 7.5%, 3.2% and 1.2% of BMI variance in QFS discovery, QFS validation, and FAS replication samples, respectively.

## Discussion

The cumulative genetic effect of previously identified BMI-associated SNPs on obesity prevalence in the form of a polygenic score was evaluated in the present study. A continuous and weighed PRS was constructed with 231 SNPs previously reported as BMI-associated at genome-wide level. The main results highlight the potential and limitations of using the genetic background in the stratification of obesity.

To our knowledge, this is one of the BMI-associated polygenic scores with a better ability (AUC_adj_ = 0.704 in the QFS discovery sample) to stratify obesity *per se* (Loos and Janssens, 2017). Nevertheless, although the evidence of association observed in the QFS discovery sample between PRS_231_ and obesity prevalence was supported by significant results in the QFS validation sample, these results were only partially replicated in the independent FAS study. According to Dudbridge, a larger training sample size would have been needed with such limited QFS and FAS target samples (Dudbridge, 2013). However, although the size of the discovery sample is more critical than that of target samples in a polygenic score analysis (Dudbridge, 2013), we acknowledge that this study lacks of sufficient statistical power to address clinical questions in a confident manner and that a number of reported associations may be spurious. In order to maximize statistical power to overcome this issue (Dudbridge, 2013) and taking into account recent evidence suggesting that heritability of complex traits comes from large numbers of commons SNPs (Chatterjee et al., 2013; Khera et al., 2018), the polygenic score was constructed herein with all the 231 available obesity-associated SNPs and not only with the 31 significantly associated SNPs. In this regard, the use of larger numbers of GWAS markers, combined with adequate sample sizes, represent a promising strategy when approaching the development of genetic tools focused on disease stratification (Wray et al., 2013). Although it would be preferable that SNP weights had been derived from independent datasets, the heterogeneity in effect size calculations among studies, together with the specific family-based data used in the present study, led us to derive our own SNP weights in the QFS discovery sample.

The QFS cohort involves participants from 222 French-Canadian families from Quebec City, making up a largely homogeneous ancestry population. Family relatedness was handled by using generalized linear mixed models, a statistical method successfully applied in the past when testing genetic associations in samples with family or cryptic relatedness among individuals (Choquette et al., 2012; Plourde et al., 2013; Rudkowska et al., 2015; Chen et al., 2016). A recent study comparing distributions of polygenic scores of type 2 diabetes and cardiovascular disease within populations with different ancestries has shown that the risk level estimated for one population can considerably differ from the level in another (Reisberg et al., 2017). Accordingly, another study focused on the contribution of polygenic risk to obesity reported different effects of the genetic score on BMI across different ethnics and birth cohorts (Walter et al., 2016). Herein, the cumulative effect of 231 SNPs resulted in a difference of more than 6.5 BMI units (31.5 vs 25 kg/m^2^) between subjects in the fifth and first PRS_231_ quintiles in the QFS discovery sample, and 4.5 BMI units (30.0 vs 25.5 kg/m^2^) in the QFS discovery sample. Although main results were consistent across quintiles in the independent and more heterogenous FAS study, we acknowledge that the ethnic homogeneity of QFS may represent a limitation to the generalizability of the results, and larger studies are still required for accurately testing the clinical utility of a polygenic score to stratify obesity.

On the other hand, compared to previous GWAS results, where reported single-SNP associations with obesity explained less than 1% of BMI variance (Frayling et al., 2007; Loos et al., 2008), polygenic scores are able to progressively increase the proportion of variance accounted for by using cumulative series of BMI-associated SNPs (Speliotes et al., 2010; Hung et al., 2015; Locke et al., 2015). In this regard, PRS_231_ is to date the polygenic score involving the largest number of BMI-associated SNPs and had a cumulative impact on BMI accounting for 7.5% and 3.2% of phenotypic variance in both QFS discovery and replication samples, respectively. Although the proportion of the phenotypic variance attributable to genetic variance is one of the largest among previous obesity polygenic scores (Loos and Janssens, 2017), this is certainly not a large proportion of phenotypic variance if we consider that more than 90% of the variance is still unaccounted for. This issue, together with an insufficient statistical power driven by limited sample sizes, represent the main weaknesses of using polygenic scores to stratify complex phenotypes such as obesity.

A recent study in the QFS cohort, where a PRS with 97 BMI-associated SNPs was also developed, showed that eating behavior played an important role in the genetic susceptibility to obesity (Jacob et al., 2018). Similarly, other studies have reported a significant impact of satiety mechanisms (Llewellyn et al., 2014) or fat and energy intake (Celis-Morales et al., 2017) on the genetic susceptibility to obesity assessed from genetic scores (28 and 93 BMI-associated SNPs, respectively). In agreement, previous studies suggesting a great impact of genetic background in body weight loss after bariatric surgery (Rinella et al., 2013; Moore et al., 2014; de Toro-Martín et al., 2018) also point to the need of focusing on the interaction between genetic background with other factors influencing weight loss outcomes. Future research in the field is expected to boost the accuracy and reliability of PRSs in anticipating the onset of metabolic diseases, such as obesity, leading to an early management of such disorders (Khera et al., 2019). Altogether, these results highlight the relevance of accurately identifying all the factors involved in obesity development and body weight management, as well as their interaction with the genetic background, for a better disease stratification. Deepen on these factors and on their relationship with each other will help on the accurate identification of obesity-prone individuals, who may benefit more from precision nutrition or lifestyle interventions.

In conclusion, in the present study, a generalized linear mixed model was fit in order to stratify obesity prevalence by means of a polygenic score. Main results revealed that genetic background in the form of a 231 BMI-associated PRS has a cumulative impact on obesity, but a limited potential to accurately stratify it. These results should be then taken with caution, as the ability of this polygenic score in classifying obesity status is not accurate enough at the individual patient level. Further studies are encouraged on larger samples with more comprehensive genetic scores.

## Ethics Statement

Experimental protocols of both QFS and FAS studies were approved by the ethic committee of Laval University and were conducted in accordance with the Declaration of Helsinki. Participants of both studies provided written informed consent.

## Author Contributions

JT-M performed statistical analysis, interpreted the data, and wrote the manuscript. M-CV and FG conceived and designed the research. CB, AT, and LP participated in development and implementation of the QFS study, and critically reviewed the manuscript.

## Funding

This study was supported by a grant-in-aid from the Heart and Stroke Foundation of Canada (G-17-0016627) and by the Canada Research Chair in Genomics Applied to Nutrition and Metabolic Health held by M-CV. JT-M received a postdoctoral fellowship from the Fonds de Recherche du Québec-Santé.

## Conflict of Interest

The authors declare that the research was conducted in the absence of any commercial or financial relationships that could be construed as a potential conflict of interest.

## References

[B1] Ashwell M. Cole T. J. Dixon A. K. (1985). Obesity: new insight into the anthropometric classification of fat distribution shown by computed tomography. Br. Med. J. (Clin. Res. Ed.) 290, 1692–1694. 10.1136/bmj.290.6483.1692 PMC1416121 3924217

[B2] Celis-Morales C. A. Lyall D. M. Gray S. R. Steell L. Anderson J. Iliodromiti S. (2017). Dietary fat and total energy intake modifies the association of genetic profile risk score on obesity: evidence from 48 170 UK Biobank participants. Int. J. Obes. 41, 1761–1768. 10.1038/ijo.2017.169 28736445

[B3] Chang C. C. Chow C. C. Tellier L. C. Vattikuti S. Purcell S. M. Lee J. J. (2015). Second-generation PLINK: rising to the challenge of larger and richer datasets. Gigascience 4, 7. 10.1186/s13742-015-0047-8 25722852 PMC4342193

[B4] Chaput J.-P. Pérusse L. Després J.-P. Tremblay A. Bouchard C. (2014). Findings from the Quebec family study on the etiology of obesity: genetics and environmental highlights. Curr. Obes. Rep. 3, 54–66. 10.1007/s13679-013-0086-3 24533236 PMC3920031

[B5] Chatterjee N. Wheeler B. Sampson J. Hartge P. Chanock S. J. Park J. H. (2013). Projecting the performance of risk prediction based on polygenic analyses of genome-wide association studies. Nat. Genet. 45, 400–405. 10.1038/ng.2579 23455638 PMC3729116

[B6] Chen H. Wang C. Conomos M. P. Stilp A. M. Li Z. Sofer T. (2016). Control for population structure and relatedness for binary traits in genetic association studies via logistic mixed models. Am. J. Hum. Genet. 98, 653–666. 10.1016/j.ajhg.2016.02.012 27018471 PMC4833218

[B7] Chen M.-H. Yang Q. (2010). GWAF: an R package for genome-wide association analyses with family data. Bioinformatics 26, 580–581. 10.1093/bioinformatics/btp710 20040588 PMC2852219

[B8] Choquette A. C. Bouchard L. Drapeau V. Lemieux S. Tremblay A. Bouchard C. (2012). Association between olfactory receptor genes, eating behavior traits and adiposity: results from the Quebec Family Study. Physiol. Behav. 105, 772–776. 10.1016/j.physbeh.2011.10.015 22044667

[B9] Das S. Forer L. Schönherr S. Sidore C. Locke A. E. Kwong A. (2016). Next-generation genotype imputation service and methods. Nat. Genet. 48, 1284–1287. 10.1038/ng.3656 27571263 PMC5157836

[B10] de Toro-Martín J. Guénard F. Tchernof A. Pérusse L. Marceau S. Vohl M.-C. (2018). Polygenic risk score for predicting weight loss after bariatric surgery. JCI Insight 3, e122011. 10.1172/jci.insight.122011 PMC6171810 30185664

[B11] Do R. Bailey S. D. Desbiens K. Belisle A. Montpetit A. Bouchard C. (2008). Genetic variants of FTO influence adiposity, insulin sensitivity, leptin levels, and resting metabolic rate in the Quebec family study. Diabetes 57, 1147–1150. 10.2337/db07-1267 18316358

[B12] Dudbridge F. (2013). Power and predictive accuracy of polygenic risk scores. PLoS Genet. 9, e1003348. 10.1371/journal.pgen.1003348 23555274 PMC3605113

[B13] Frayling T. M. Timpson N. J. Weedon M. N. Zeggini E. Freathy R. M. Lindgren C. M. (2007). A common variant in the FTO gene is associated with body mass index and predisposes to childhood and adult obesity. Science 316, 889–894. 10.1126/science.1141634 17434869 PMC2646098

[B14] Ghosh S. Bouchard C. (2017). Convergence between biological, behavioural and genetic determinants of obesity. Nat. Rev. Genet. 18, 731–748. 10.1038/nrg.2017.72 28989171

[B15] Harrell F. E. (2018). Hmisc: Harrell Miscellaneous. R package version 4.1-1. https://CRAN.R-project.org/package=Hmisc

[B16] Hung C.-F. Breen G. Czamara D. Corre T. Wolf C. Kloiber S. (2015). A genetic risk score combining 32 SNPs is associated with body mass index and improves obesity prediction in people with major depressive disorder. BMC Med. 13, 86. 10.1186/s12916-015-0334-3 25903154 PMC4407390

[B17] Jacob R. Drapeau V. Tremblay A. Provencher V. Bouchard C. Pérusse L. (2018). The role of eating behavior traits in mediating genetic susceptibility to obesity. Am. J. Clin. Nutr. 108, 445–452. 10.1093/ajcn/nqy130 29982344

[B18] Khera A. V. Chaffin M. Aragam K. G. Haas M. E. Roselli C. Choi S. H. (2018). Genome-wide polygenic scores for common diseases identify individuals with risk equivalent to monogenic mutations. Nat. Genet. 50, 1219. 10.1038/s41588-018-0183-z 30104762 PMC6128408

[B19] Khera A. V. Chaffin M. Wade K. H. Zahid S. Brancale J. Xia R. (2019). Polygenic prediction of weight and obesity trajectories from birth to adulthood. Cell 177, 587–596.e9. 10.1016/j.cell.2019.03.028 31002795 PMC6661115

[B20] Lean M. E. Han T. S. Morrison C. E. (1995). Waist circumference as a measure for indicating need for weight management. BMJ 311, 158–161. 10.1136/bmj.311.6998.158 7613427 PMC2550221

[B21] Li S. Zhao J. H. Luan J. Luben R. N. Rodwell S. A. Khaw K.-T. (2010a). Cumulative effects and predictive value of common obesity-susceptibility variants identified by genome-wide association studies. Am. J. Clin. Nutr. 91, 184–190. 10.3945/ajcn.2009.28403 19812171

[B22] Li Y. Willer C. J. Ding J. Scheet P. Abecasis G. R. (2010b). MaCH: using sequence and genotype data to estimate haplotypes and unobserved genotypes. Genet. Epidemiol. 34, 816–834. 10.1002/gepi.20533 21058334 PMC3175618

[B23] Llewellyn C. H. Trzaskowski M. van Jaarsveld C. H. M. Plomin R. Wardle J. (2014). Satiety mechanisms in genetic risk of obesity. JAMA Pediatr. 168, 338. 10.1001/jamapediatrics.2013.4944 24535189 PMC3981891

[B24] Locke A. E. Kahali B. Berndt S. I. Justice A. E. Pers T. H. Day F. R. (2015). Genetic studies of body mass index yield new insights for obesity biology. Nature 518, 197–206. 10.1038/nature14177 25673413 PMC4382211

[B25] Loos R. J. F. Janssens A. C. J. W. (2017). predicting polygenic obesity using genetic information. Cell Metab. 25, 535–543. 10.1016/j.cmet.2017.02.013 28273476

[B26] Loos R. J. F. Lindgren C. M. Li S. Wheeler E. Zhao J. H. Prokopenko I. (2008). Common variants near MC4R are associated with fat mass, weight and risk of obesity. Nat. Genet. 40, 768–775. 10.1038/ng.140 18454148 PMC2669167

[B27] Moore B. S. Mirshahi U. L. Yost E. A. Stepanchick A. N. Bedrin M. D. Styer A. M. (2014). Long-term weight-loss in gastric bypass patients carrying melanocortin 4 receptor variants. PLoS One 9, e93629. 10.1371/journal.pone.0093629 24705671 PMC3976318

[B28] Palla L. Dudbridge F. (2015). A fast method that uses polygenic scores to estimate the variance explained by genome-wide marker panels and the proportion of variants affecting a trait. Am. J. Hum. Genet. 97, 250–259. 10.1016/j.ajhg.2015.06.005 26189816 PMC4573448

[B29] Pencina M. J. D’Agostino R. B. D’Agostino R. B. Vasan R. S. (2008). Evaluating the added predictive ability of a new marker: from area under the ROC curve to reclassification and beyond. Stat. Med. 27, 157–172. 10.1002/sim.2929 17569110

[B30] Peterson R. E. Maes H. H. Holmans P. Sanders A. R. Levinson D. F. Shi J. (2011). Genetic risk sum score comprised of common polygenic variation is associated with body mass index. Hum Genet. 129, 221–230. 10.1007/s00439-010-0917-1 21104096 PMC3403709

[B31] Plourde M. Vohl M.-C. Bellis C. Carless M. Dyer T. Dolley G. (2013). A variant in the *LRRFIP1* gene is associated with adiposity and inflammation. Obesity 21, 185–192. 10.1002/oby.20242 23505185

[B32] R Core Team (2018). R: a language and environment for statistical computing. Vienna, Austria: R Foundation for Statistical Computing https://www.R-project.org/

[B33] Reisberg S. Iljasenko T. Läll K. Fischer K. Vilo J. (2017). Comparing distributions of polygenic risk scores of type 2 diabetes and coronary heart disease within different populations. PLoS One 12, e0179238. 10.1371/journal.pone.0179238 28678847 PMC5497939

[B34] Rinella E. S. Still C. Shao Y. Wood G. C. Chu X. Salerno B. (2013). Genome-wide association of single-nucleotide polymorphisms with weight loss outcomes after roux-en-y gastric bypass surgery. J. Clin. Endocrinol. Metab. 98, E1131–E1136. 10.1210/jc.2012-3421 23633212 PMC3667258

[B35] Robitaille J. Després J.-P. Pérusse L. Vohl M.-C. (2003). The PPAR-gamma P12A polymorphism modulates the relationship between dietary fat intake and components of the metabolic syndrome: results from the Québec Family Study. Clin. Genet. 63, 109–116. 10.1034/j.1399-0004.2003.00026.x 12630956

[B36] Rouskas K. Kouvatsi A. Paletas K. Papazoglou D. Tsapas A. Lobbens S. (2012). Common variants in FTO, MC4R, TMEM18, PRL, AIF1, and PCSK1 show evidence of association with adult obesity in the greek population. Obesity 20, 389–395. 10.1038/oby.2011.177 21720444

[B37] Rudkowska I. Guenard F. Julien P. Couture P. Lemieux S. Barbier O. (2014). Genome-wide association study of the plasma triglyceride response to an n-3 polyunsaturated fatty acid supplementation. J. Lipid. Res. 55, 1245–1253. 10.1194/jlr.M045898 24847101 PMC4076081

[B38] Rudkowska I. Pérusse L. Bellis C. Blangero J. Després J.-P. Bouchard C. (2015). Interaction between common genetic variants and total fat intake on low-density lipoprotein peak particle diameter: a genome-wide association study. J. Nutrigenet. Nutrigenomics 8, 44–53. 10.1159/000431151 26112879

[B39] Sandholt C. H. Sparso T. Grarup N. Albrechtsen A. Almind K. Hansen L. (2010). Combined analyses of 20 common obesity susceptibility variants. Diabetes 59, 1667–1673. 10.2337/db09-1042 20110568 PMC2889766

[B40] Seidell J. C. Flegal K. M. (1997). Assessing obesity: classification and epidemiology. Br. Med. Bull. 53, 238–252. 10.1093/oxfordjournals.bmb.a011611 9246834

[B41] Speliotes E. K. Willer C. J. Berndt S. I. Monda K. L. Thorleifsson G. Jackson A. U. (2010). Association analyses of 249,796 individuals reveal 18 new loci associated with body mass index. Nat. Genet. 42, 937–948. 10.1038/ng.686 20935630 PMC3014648

[B42] Sung Y. J. Pérusse L. Sarzynski M. A. Fornage M. Sidney S. Sternfeld B. (2016). Genome-wide association studies suggest sex-specific loci associated with abdominal and visceral fat. Int. J. Obes. 40, 662–674. 10.1038/ijo.2015.217 PMC4821694 26480920

[B43] Walter S. Mejía-Guevara I. Estrada K. Liu S. Y. Glymour M. M. (2016). Association of a genetic risk score with body mass index across different birth cohorts. JAMA 316, 63. 10.1001/jama.2016.8729 27380344

[B44] Wray N. R. Yang J. Hayes B. J. Price A. L. Goddard M. E. Visscher P. M. (2013). Pitfalls of predicting complex traits from SNPs. Nat. Rev. Genet. 14, 507–515. 10.1038/nrg3457 23774735 PMC4096801

[B45] Ziyatdinov A. Vázquez-Santiago M. Brunel H. Martinez-Perez A. Aschard H. Soria J. M. (2018). lme4qtl: linear mixed models with flexible covariance structure for genetic studies of related individuals. BMC Bioinformatics 19, 68. 10.1186/s12859-018-2057-x 29486711 PMC5830078

