# Supplementary material for: The Challenge of Stratifying Obesity: Attempts in the Quebec Family Study
Source: Front Genet. 2019 Oct 10;10:994. doi: 10.3389/fgene.2019.00994 (PMC6796792; doi:10.3389/fgene.2019.00994)
Supplement: Supplementary file 1 [file DataSheet_1.pdf]

*Supplementary Material*

# The Challenge of Stratifying Obesity: Attempts in the Quebec Family Study

**Juan de Toro-Martín[1,2], Frédéric Guénard[1,2], Claude Bouchard[3], Angelo Tremblay[4,5], Louis Pérusse[1,4], Marie-Claude Vohl[1,2]***

*** Correspondence:** Dr. Marie-Claude Vohl: Marie-Claude.Vohl@fsaa.ulaval.ca

**Table S1. List of 231 BMI-associated SNPs from the NHGRI-EBI GWAS catalog.**

| SNP | Minor allele | Major allele | MAF | HWE P-value | GWAS P-value | Ref |
|---|---|---|---|---|---|---|
| rs1000940 | G | A | 0.32 | 0.70 | 1.3x10-08 | 1 |
| rs10132280 | A | C | 0.29 | 0.22 | 1.1x10-11 | 1 |
| rs1016287 | T | C | 0.26 | 1.00 | 2.3x10-11 | 1 |
| rs10182181 | G | A | 0.44 | 0.42 | 8.8x10-24 | 1 |
| rs10733682 | A | G | 0.46 | 0.04 | 1.8x10-08 | 1 |
| rs10938397 | G | A | 0.44 | 0.65 | 3.2x10-38 | 1 |
| rs10968576 | G | A | 0.34 | 0.45 | 6.6x10-14 | 1 |
| rs11030104 | G | A | 0.23 | 0.52 | 5.6x10-28 | 1 |
| rs11057405 | A | G | 0.09 | 0.16 | 2.0x10-08 | 1 |
| rs11126666 | A | G | 0.23 | 0.87 | 1.3x10-09 | 1 |
| rs11165643 | C | T | 0.42 | 0.82 | 2.1x10-12 | 1 |
| rs11170468 | C | A | 0.28 | 0.10 | 7.0x10-08 | 1 |
| rs11191560 | C | T | 0.08 | 0.25 | 8.4x10-09 | 1 |
| rs11583200 | C | T | 0.41 | 0.56 | 1.5x10-08 | 1 |
| rs1167827 | A | G | 0.40 | 0.64 | 6.3x10-10 | 1 |
| rs11688816 | A | G | 0.47 | 0.05 | 1.9x10-08 | 1 |
| rs11727676 | C | T | 0.04 | 1.00 | 2.6x10-08 | 1 |
| rs11847697 | T | C | 0.05 | 0.56 | 4.0x10-09 | 1 |
| rs12286929 | A | G | 0.45 | 0.57 | 1.3x10-12 | 1 |
| rs12401738 | A | G | 0.33 | 0.90 | 1.1x10-10 | 1 |
| rs12429545 | A | G | 0.13 | 0.02 | 1.1x10-12 | 1 |
| rs12446632 | A | G | 0.17 | 0.32 | 1.5x10-18 | 1 |
| rs12566985 | G | A | 0.46 | 0.36 | 3.3x10-15 | 1 |
| rs12885454 | A | C | 0.34 | 0.53 | 1.9x10-10 | 1 |
| rs12940622 | A | G | 0.45 | 0.30 | 2.5x10-09 | 1 |
| rs13021737 | A | G | 0.18 | 0.70 | 1.1x10-50 | 1 |
| rs13078960 | G | T | 0.23 | 1.00 | 1.7x10-14 | 1 |
| rs13107325 | T | C | 0.08 | 0.71 | 1.8x10-12 | 1 |
| rs13191362 | G | A | 0.14 | 0.36 | 7.3x10-09 | 1 |
| rs13201877 | G | A | 0.15 | 0.08 | 4.3x10-08 | 1 |
| rs1441264 | G | A | 0.44 | 0.82 | 3.0x10-08 | 1 |
| rs1460676 | C | T | 0.13 | 0.63 | 5.0x10-08 | 1 |
| rs1516725 | T | C | 0.13 | 0.80 | 1.9x10-22 | 1 |
| rs1528435 | C | T | 0.39 | 0.41 | 1.2x10-08 | 1 |
| rs1558902 | A | T | 0.40 | 0.56 | 7.5x10-153 | 1 |
| rs16851483 | T | G | 0.06 | 1.00 | 3.5x10-10 | 1 |
| rs16907751 | T | C | 0.11 | 0.78 | 3.9x10-08 | 1 |
| rs16951275 | C | T | 0.23 | 0.20 | 1.9x10-17 | 1 |
| rs17001654 | G | C | 0.19 | 0.85 | 7.8x10-09 | 1 |
| rs17024393 | C | T | 0.02 | 1.00 | 7.0x10-14 | 1 |
| rs17094222 | C | T | 0.20 | 0.59 | 5.9x10-11 | 1 |
| rs17203016 | G | A | 0.19 | 0.58 | 3.4x10-08 | 1 |
| rs17405819 | C | T | 0.29 | 0.33 | 2.1x10-11 | 1 |
| rs17724992 | G | A | 0.27 | 1.00 | 3.4x10-08 | 1 |
| rs1808579 | T | C | 0.45 | 0.91 | 4.2x10-08 | 1 |
| rs1928295 | C | T | 0.45 | 0.36 | 7.9x10-10 | 1 |
| rs2033529 | G | A | 0.26 | 0.88 | 1.4x10-08 | 1 |
| rs2033732 | T | C | 0.25 | 0.65 | 4.9x10-08 | 1 |
| rs205262 | G | A | 0.27 | 0.31 | 1.8x10-10 | 1 |
| rs2075650 | G | A | 0.10 | 0.75 | 1.2x10-08 | 1 |
| rs2080454 | C | A | 0.34 | 1.00 | 8.6x10-09 | 1 |
| rs2112347 | G | T | 0.37 | 1.00 | 6.2x10-17 | 1 |
| rs2121279 | T | C | 0.09 | 1.00 | 2.3x10-08 | 1 |
| rs2176040 | A | G | 0.36 | 0.46 | 1.0x10-08 | 1 |
| rs2176598 | T | C | 0.29 | 0.49 | 3.0x10-08 | 1 |
| rs2207139 | G | A | 0.14 | 0.82 | 4.1x10-29 | 1 |
| rs2287019 | T | C | 0.15 | 0.38 | 4.6x10-18 | 1 |
| rs2365389 | T | C | 0.44 | 0.49 | 1.6x10-10 | 1 |
| rs2650492 | A | G | 0.28 | 0.89 | 1.9x10-09 | 1 |
| rs2820292 | A | C | 0.42 | 0.25 | 1.8x10-10 | 1 |
| rs2836754 | T | C | 0.37 | 0.54 | 1.6x10-08 | 1 |
| rs29941 | A | G | 0.33 | 0.90 | 2.4x10-08 | 1 |
| rs3101336 | T | C | 0.33 | 0.80 | 2.7x10-26 | 1 |
| rs3736485 | A | G | 0.43 | 0.21 | 7.4x10-09 | 1 |
| rs3810291 | G | A | 0.32 | 0.44 | 4.8x10-15 | 1 |
| rs3817334 | T | C | 0.45 | 1.00 | 5.1x10-17 | 1 |

| | | | | | | |
|---|---|---|---|---|---|---|
| rs3849570 | A | C | 0.33 | 0.44 | 2.6x10-08 | 1 |
| rs3888190 | A | C | 0.36 | 0.81 | 3.1x10-23 | 1 |
| rs4256980 | C | G | 0.37 | 0.47 | 2.9x10-11 | 1 |
| rs4740619 | C | T | 0.42 | 0.56 | 4.6x10-09 | 1 |
| rs4787491 | A | G | 0.48 | 1.00 | 2.7x10-08 | 1 |
| rs492400 | C | T | 0.40 | 0.56 | 6.8x10-09 | 1 |
| rs543874 | G | A | 0.21 | 0.87 | 2.6x10-35 | 1 |
| rs6091540 | T | C | 0.27 | 0.78 | 2.2x10-11 | 1 |
| rs6465468 | T | G | 0.36 | 0.81 | 5.0x10-08 | 1 |
| rs6477694 | C | T | 0.33 | 0.52 | 2.7x10-08 | 1 |
| rs6567160 | C | T | 0.27 | 1.00 | 3.9x10-53 | 1 |
| rs657452 | A | G | 0.40 | 0.16 | 5.5x10-13 | 1 |
| rs6804842 | A | G | 0.42 | 0.82 | 2.5x10-09 | 1 |
| rs7138803 | A | G | 0.38 | 0.55 | 8.2x10-24 | 1 |
| rs7141420 | C | T | 0.48 | 0.21 | 1.2x10-14 | 1 |
| rs7164727 | C | T | 0.31 | 0.90 | 3.9x10-09 | 1 |
| rs7239883 | G | A | 0.41 | 0.24 | 1.5x10-08 | 1 |
| rs7243357 | G | T | 0.16 | 0.14 | 3.9x10-08 | 1 |
| rs758747 | T | C | 0.33 | 1.00 | 7.5x10-10 | 1 |
| rs7599312 | A | G | 0.27 | 0.78 | 1.2x10-10 | 1 |
| rs7715256 | G | T | 0.40 | 0.24 | 8.9x10-09 | 1 |
| rs7899106 | G | A | 0.04 | 1.00 | 3.0x10-08 | 1 |
| rs7903146 | T | C | 0.33 | 1.00 | 1.1x10-11 | 1 |
| rs9374842 | C | T | 0.26 | 0.46 | 2.7x10-08 | 1 |
| rs9400239 | T | C | 0.35 | 0.80 | 1.6x10-08 | 1 |
| rs9540493 | A | G | 0.42 | 0.91 | 5.0x10-08 | 1 |
| rs9641123 | C | G | 0.42 | 0.30 | 2.1x10-10 | 1 |
| rs977747 | T | G | 0.35 | 1.00 | 2.2x10-08 | 1 |
| rs9914578 | G | C | 0.18 | 0.71 | 2.1x10-08 | 1 |
| rs9925964 | G | A | 0.40 | 0.41 | 8.1x10-10 | 1 |
| rs1406503 | C | G | 0.03 | 1.00 | 9.0x10-06 | 2 |
| rs1440072 | C | T | 0.06 | 1.00 | 4.0x10-06 | 2 |
| rs17124318 | G | C | 0.03 | 0.20 | 6.0x10-07 | 2 |
| rs2373011 | G | C | 0.45 | 0.65 | 9.0x10-06 | 2 |
| rs817858 | G | C | 0.02 | 0.14 | 7.0x10-06 | 2 |
| rs9906155 | C | T | 0.06 | 0.36 | 9.0x10-06 | 2 |
| rs1106683 | A | G | 0.12 | 0.29 | 1.0x10-07 | 3 |
| rs1106684 | G | C | 0.12 | 0.29 | 2.0x10-06 | 3 |
| rs1333026 | A | G | 0.17 | 1.00 | 8.0x10-06 | 3 |
| rs10789336 | G | A | 0.33 | 0.70 | 1.0x10-06 | 4 |
| rs13130484 | T | C | 0.44 | 0.73 | 6.0x10-09 | 4 |
| rs1321847 | A | G | 0.11 | 1.00 | 2.0x10-06 | 4 |
| rs1514175 | A | G | 0.44 | 0.42 | 3.0x10-11 | 4 |
| rs1561288 | T | C | 0.22 | 1.00 | 5.0x10-08 | 4 |
| rs2236835 | A | G | 0.04 | 1.00 | 8.0x10-06 | 4 |
| rs3936060 | G | C | 0.18 | 1.00 | 5.0x10-06 | 4 |
| rs6107853 | A | G | 0.43 | 0.49 | 2.0x10-06 | 4 |
| rs7234864 | T | C | 0.29 | 0.41 | 4.0x10-17 | 4 |
| rs8005845 | C | T | 0.25 | 0.45 | 5.0x10-06 | 4 |
| rs9940128 | A | G | 0.41 | 0.56 | 4.0x10-23 | 4 |
| rs1024889 | G | A | 0.29 | 0.89 | 6.0x10-06 | 5 |
| rs1152846 | T | C | 0.25 | 0.18 | 3.0x10-06 | 5 |
| rs12517906 | T | C | 0.22 | 0.14 | 6.0x10-06 | 5 |
| rs1458095 | T | C | 0.07 | 0.68 | 7.0x10-06 | 5 |
| rs1878047 | G | A | 0.36 | 0.11 | 5.0x10-06 | 5 |
| rs1927702 | C | T | 0.46 | 0.91 | 6.0x10-06 | 5 |
| rs2383393 | A | G | 0.36 | 0.81 | 2.0x10-06 | 5 |
| rs3803915 | A | C | 0.10 | 0.19 | 5.0x10-06 | 5 |
| rs3934834 | T | C | 0.13 | 0.46 | 6.0x10-07 | 5 |
| rs824931 | C | T | 0.37 | 0.90 | 3.0x10-06 | 5 |
| rs10458787 | A | G | 0.22 | 0.87 | 1.0x10-06 | 6 |
| rs2275215 | C | T | 0.31 | 1.00 | 4.0x10-07 | 6 |
| rs1121980 | A | G | 0.41 | 0.64 | 4.0x10-08 | 7 |
| rs10136789 | C | T | 0.11 | 0.76 | 5.0x10-07 | 8 |
| rs10889850 | T | G | 0.39 | 0.81 | 2.0x10-06 | 8 |
| rs12693973 | G | T | 0.28 | 0.33 | 2.0x10-06 | 8 |
| rs12964056 | A | G | 0.29 | 0.41 | 7.0x10-07 | 8 |
| rs1542829 | A | G | 0.06 | 0.10 | 1.0x10-08 | 8 |
| rs2016586 | T | G | 0.36 | 0.11 | 3.0x10-06 | 8 |
| rs8050136 | A | C | 0.38 | 0.47 | 1.0x10-07 | 8 |
| rs8192472 | T | C | 0.37 | 0.27 | 1.0x10-06 | 8 |
| rs2033195 | C | T | 0.40 | 0.16 | 6.0x10-06 | 9 |

| | | | | | | |
|---|---|---|---|---|---|---|
| rs6794092 | A | G | 0.02 | 1.00 | 2.0x10-06 | 9 |
| rs10993160 | G | A | 0.03 | 1.00 | 6.0x10-07 | 10 |
| rs12149832 | A | G | 0.40 | 0.24 | 5.0x10-22 | 10 |
| rs13034723 | A | G | 0.43 | 0.42 | 2.0x10-08 | 10 |
| rs2206734 | T | C | 0.15 | 0.67 | 1.0x10-11 | 10 |
| rs2331841 | A | G | 0.47 | 0.73 | 2.0x10-11 | 10 |
| rs4377469 | G | T | 0.11 | 1.00 | 2.0x10-07 | 10 |
| rs516636 | T | G | 0.20 | 0.86 | 3.0x10-09 | 10 |
| rs3096490 | A | G | 0.37 | 0.33 | 5.0x10-07 | 11 |
| rs955423 | C | A | 0.42 | 1.00 | 3.0x10-07 | 11 |
| rs10150332 | C | T | 0.21 | 0.32 | 3.0x10-11 | 12 |
| rs10767664 | T | A | 0.23 | 0.26 | 5.0x10-26 | 12 |
| rs12444979 | T | C | 0.17 | 0.32 | 3.0x10-21 | 12 |
| rs13078807 | G | A | 0.23 | 0.88 | 4.0x10-11 | 12 |
| rs1555543 | A | C | 0.42 | 0.64 | 4.0x10-10 | 12 |
| rs206936 | G | A | 0.21 | 0.39 | 3.0x10-08 | 12 |
| rs2241423 | A | G | 0.22 | 0.25 | 1.0x10-18 | 12 |
| rs2444217 | G | A | 0.39 | 0.72 | 9.0x10-08 | 12 |
| rs255414 | G | A | 0.18 | 0.57 | 1.0x10-06 | 12 |
| rs2815752 | G | A | 0.33 | 0.80 | 2.0x10-22 | 12 |
| rs2867125 | T | C | 0.18 | 1.00 | 3.0x10-49 | 12 |
| rs2890652 | C | T | 0.13 | 0.81 | 1.0x10-10 | 12 |
| rs2922763 | G | T | 0.26 | 0.77 | 6.0x10-08 | 12 |
| rs3764400 | C | T | 0.14 | 0.33 | 4.0x10-07 | 12 |
| rs4771122 | G | A | 0.29 | 0.22 | 9.0x10-10 | 12 |
| rs4836133 | C | A | 0.47 | 0.65 | 2.0x10-09 | 12 |
| rs4929949 | C | T | 0.49 | 0.74 | 3.0x10-09 | 12 |
| rs571312 | A | C | 0.27 | 1.00 | 6.0x10-42 | 12 |
| rs713586 | C | T | 0.43 | 0.57 | 6.0x10-22 | 12 |
| rs7359397 | T | C | 0.36 | 0.81 | 2.0x10-20 | 12 |
| rs867559 | G | A | 0.17 | 0.84 | 1.0x10-07 | 12 |
| rs887912 | T | C | 0.26 | 0.88 | 2.0x10-12 | 12 |
| rs9816226 | A | T | 0.18 | 0.56 | 2.0x10-18 | 12 |
| rs10783050 | C | T | 0.38 | 0.90 | 4.0x10-06 | 13 |
| rs10913469 | C | T | 0.21 | 0.49 | 6.0x10-08 | 13 |
| rs12970134 | A | G | 0.29 | 0.68 | 1.0x10-12 | 13 |
| rs6265 | T | C | 0.21 | 0.74 | 5.0x10-10 | 13 |
| rs6499640 | G | A | 0.42 | 0.56 | 4.0x10-13 | 13 |
| rs7481311 | T | C | 0.23 | 0.27 | 8.0x10-06 | 13 |
| rs7561317 | A | G | 0.17 | 0.84 | 4.0x10-17 | 13 |
| rs7647305 | T | C | 0.21 | 1.00 | 7.0x10-11 | 13 |
| rs925946 | T | G | 0.30 | 0.79 | 9.0x10-10 | 13 |
| rs10769908 | C | T | 0.49 | 0.74 | 1.0x10-06 | 14 |
| rs10838738 | G | A | 0.36 | 0.62 | 5.0x10-09 | 14 |
| rs11084753 | A | G | 0.34 | 1.00 | 2.0x10-08 | 14 |
| rs12324805 | C | A | 0.31 | 0.59 | 7.0x10-06 | 14 |
| rs17782313 | C | T | 0.27 | 0.78 | 5.0x10-18 | 14 |
| rs2145270 | C | T | 0.35 | 0.90 | 6.0x10-06 | 14 |
| rs6548238 | T | C | 0.18 | 1.00 | 1.0x10-18 | 14 |
| rs7498665 | G | A | 0.36 | 0.81 | 5.0x10-11 | 14 |
| rs9939609 | A | T | 0.38 | 0.23 | 4.0x10-51 | 14 |
| rs10904363 | C | G | 0.05 | 1.00 | 3.0x10-06 | 15 |
| rs4432245 | C | T | 0.05 | 0.49 | 1.0x10-06 | 15 |
| rs7202116 | G | A | 0.38 | 0.23 | 2.0x10-10 | 16 |
| rs11075990 | G | A | 0.38 | 0.23 | 2.0x10-51 | 17 |
| rs2030323 | A | C | 0.23 | 0.26 | 6.0x10-10 | 17 |
| rs2568958 | G | A | 0.33 | 0.80 | 2.0x10-14 | 17 |
| rs2903492 | G | A | 0.18 | 0.70 | 6.0x10-15 | 17 |
| rs633715 | C | T | 0.20 | 1.00 | 5.0x10-12 | 17 |
| rs8089364 | C | T | 0.30 | 0.69 | 4.0x10-21 | 17 |
| rs987237 | G | A | 0.16 | 1.00 | 2.0x10-11 | 17 |
| rs10261878 | A | C | 0.04 | 1.00 | 1.0x10-10 | 18 |
| rs17817964 | T | C | 0.39 | 0.19 | 1.0x10-10 | 18 |
| rs348495 | A | G | 0.41 | 0.08 | 2.0x10-10 | 18 |
| rs7586879 | T | C | 0.32 | 0.09 | 4.0x10-08 | 18 |
| rs7708584 | A | G | 0.40 | 0.24 | 5.0x10-14 | 18 |
| rs974417 | T | C | 0.14 | 1.00 | 6.0x10-06 | 18 |
| rs62033400 | G | A | 0.39 | 0.29 | 2.0x10-14 | 19 |
| rs939583 | C | T | 0.18 | 0.70 | 1.0x10-07 | 19 |
| rs2607292 | T | C | 0.10 | 0.76 | 4.0x10-06 | 20 |
| rs2967951 | T | C | 0.12 | 1.00 | 1.0x10-06 | 20 |

| | | | | | | |
|---|---|---|---|---|---|---|
| rs933117 | A | G | 0.07 | 0.39 | 6.0x10-06 | 20 |
| rs11142387 | A | C | 0.46 | 0.21 | 3.0x10-08 | 21 |
| rs11191580 | C | T | 0.08 | 0.26 | 4.0x10-08 | 21 |
| rs11671664 | A | G | 0.08 | 0.13 | 3.0x10-12 | 21 |
| rs12463617 | A | C | 0.18 | 0.70 | 2.0x10-12 | 21 |
| rs12597579 | T | C | 0.06 | 0.29 | 6.0x10-07 | 21 |
| rs16858082 | C | T | 0.41 | 0.08 | 4.0x10-09 | 21 |
| rs2237892 | T | C | 0.06 | 0.61 | 9.0x10-13 | 21 |
| rs2531995 | C | T | 0.38 | 0.06 | 7.0x10-08 | 21 |
| rs2535633 | G | C | 0.42 | 0.91 | 2.0x10-10 | 21 |
| rs261967 | C | A | 0.41 | 0.56 | 8.0x10-13 | 21 |
| rs4776970 | T | A | 0.36 | 0.46 | 3.0x10-07 | 21 |
| rs4854307 | C | T | 0.06 | 1.00 | 2.0x10-06 | 21 |
| rs574367 | T | G | 0.21 | 1.00 | 2.0x10-19 | 21 |
| rs591166 | A | T | 0.46 | 0.82 | 7.0x10-14 | 21 |
| rs6545814 | G | A | 0.42 | 0.11 | 1.0x10-10 | 21 |
| rs6893807 | G | A | 0.19 | 0.58 | 1.0x10-06 | 21 |
| rs888789 | G | A | 0.47 | 0.91 | 4.0x10-06 | 21 |
| rs9356744 | C | T | 0.29 | 0.89 | 5.0x10-13 | 21 |
| rs9473924 | T | G | 0.25 | 0.55 | 4.0x10-07 | 21 |
| rs9568867 | A | G | 0.13 | 0.02 | 4.0x10-06 | 21 |
| rs652722 | T | C | 0.23 | 0.53 | 8.0x10-08 | 22 |

A total of 163 significant BMI-associated SNPs ($P<5\times10^{-8}$) and 68 SNPs near-significant BMI-associated ($P<1\times10^{-6}$) were selected from 16 previous GWAS (1–16) and 6 GWAS meta-analysis (17–22). SNP: single nucleotide polymorphism. Ref points to the original study reference. MAF: minor allele frequency in the cohort of 881 QFS participans. HWE P-value: Hardy Weinberg equilibrium P-value. GWAS P-value: original P-value of association with BMI. Grey-shaded SNPs were excluded based on HWE or MAF criteria.

**Table S2. Association results of 231 BMI-associated SNPs to obesity in the Quebec Family Study discovery sample**

| SNP | Reported gene | Position | Minor allele | Major allele | β (SE) | $X^2$ (P-value) |
|---|---|---|---|---|---|---|
| rs1000940 | RABEP1 | 17:5223976 | G | A | -0.11 (0.16) | 0.45 (0.504) |
| rs10132280 | STXBP6 | 14:24998019 | A | C | -0.08 (0.17) | 0.23 (0.633) |
| rs10136789 | KCNH5 | 14:62889535 | C | T | 0.53 (0.24) | 4.87 (0.027) |
| rs10150332 | NRXN3 | 14:79470621 | C | T | 0.10 (0.19) | 0.32 (0.573) |
| rs1016287 | LINC01122 | 2:59159129 | T | C | 0.26 (0.17) | 2.37 (0.123) |
| rs10182181 | ADCY3 | 2:25003800 | G | A | 0.00 (0.15) | 0.00 (0.974) |
| rs1024889 | NR | 3:70414179 | G | A | 0.09 (0.16) | 0.29 (0.588) |
| rs10261878 | MIR148A | 7:25910925 | A | C | 0.19 (0.40) | 0.24 (0.626) |
| rs10458787 | intergenic | 10:4613373 | A | G | 0.04 (0.19) | 0.04 (0.851) |
| rs10733682 | LMX1B | 9:128500735 | A | G | 0.19 (0.15) | 1.57 (0.211) |
| rs10767664 | BDNF | 11:27704439 | T | A | 0.06 (0.18) | 0.10 (0.756) |
| rs10769908 | STK33 | 11:8462542 | C | T | 0.01 (0.15) | 0.01 (0.936) |
| rs10783050 | NR | 1:96571527 | C | T | 0.04 (0.16) | 0.05 (0.817) |
| rs10789336 | NEGR1 | 1:72372723 | G | A | -0.10 (0.16) | 0.37 (0.542) |
| rs10838738 | MTCH2 | 11:47641497 | G | A | 0.12 (0.15) | 0.59 (0.441) |
| rs10889850 | LRRC7 | 1:69741396 | T | G | -0.06 (0.16) | 0.13 (0.720) |
| rs10904363 | AKR1CL2 | 10:4878221 | C | G | 0.56 (0.34) | 2.69 (0.101) |
| rs10913469 | RASAL2 | 1:177944384 | C | T | -0.03 (0.19) | 0.03 (0.872) |
| rs10938397 | GNPDA2 | 4:44877284 | G | A | 0.19 (0.15) | 1.51 (0.219) |
| rs10968576 | LINGO2 | 9:28404339 | G | A | -0.37 (0.17) | 4.89 (0.027) |
| rs10993160 | ZNF169 | 9:94306644 | G | A | 0.05 (0.44) | 0.01 (0.908) |
| rs11030104 | BDNF | 11:27641093 | G | A | 0.09 (0.18) | 0.23 (0.634) |
| rs11057405 | CLIP1 | 12:121347850 | A | G | -0.27 (0.28) | 0.89 (0.345) |
| rs1106683 | intergenic | 7:131768766 | A | G | 0.07 (0.24) | 0.08 (0.776) |
| rs1106684 | intergenic | 7:131768906 | G | C | 0.07 (0.24) | 0.08 (0.776) |
| rs11075990 | FTO | 16:53785981 | G | A | 0.47 (0.15) | 9.53 (0.002) |
| rs11084753 | KCTD15 | 19:33831232 | A | G | 0.08 (0.16) | 0.24 (0.625) |
| rs11126666 | KCNK3 | 2:26782315 | A | G | 0.11 (0.18) | 0.35 (0.556) |
| rs11142387 | KLF9 | 9:70383416 | A | C | -0.01 (0.15) | 0.00 (0.972) |
| rs11165643 | PTBP2 | 1:96696685 | C | T | 0.13 (0.15) | 0.79 (0.374) |
| rs11170468 | CPNE8 | 12:37716315 | C | A | -0.17 (0.17) | 0.99 (0.319) |
| rs11191560 | NT5C2 | 10:104859028 | C | T | 0.21 (0.27) | 0.64 (0.425) |
| rs11191580 | NT5C2 | 10:103146454 | C | T | 0.20 (0.27) | 0.56 (0.455) |
| rs1121980 | FTO | 16:53775335 | A | G | 0.38 (0.15) | 6.41 (0.011) |
| rs1152846 | NR | 3:188703109 | T | C | -0.05 (0.17) | 0.10 (0.747) |
| rs11583200 | ELAVL4 | 1:50332407 | C | T | 0.15 (0.15) | 0.95 (0.330) |
| rs11671664 | GIPR | 19:45669020 | A | G | 0.00 (0.26) | 0.00 (0.989) |
| rs1167827 | HIP1 | 7:75001105 | A | G | 0.04 (0.16) | 0.06 (0.810) |
| rs11688816 | EHBP1 | 2:62906552 | A | G | -0.02 (0.15) | 0.03 (0.872) |
| rs11727676 | HHIP | 4:145878514 | C | T | 0.09 (0.36) | 0.06 (0.800) |
| rs11847697 | PRKD1 | 14:29584863 | T | C | -0.44 (0.41) | 1.13 (0.288) |
| rs12149832 | FTO | 16:53808996 | A | G | 0.52 (0.15) | 12.00 (0.001) |
| rs12286929 | CADM1 | 11:114527614 | A | G | -0.02 (0.15) | 0.02 (0.902) |
| rs12324805 | RKHD3 | 15:82059859 | C | A | 0.27 (0.16) | 2.95 (0.086) |
| rs12401738 | FUBP1 | 1:78219349 | A | G | 0.14 (0.16) | 0.76 (0.383) |
| rs12429545 | OLFM4 | 13:53000207 | A | G | -0.04 (0.22) | 0.04 (0.850) |
| rs12444979 | GPRC5B | 16:19922278 | T | C | -0.33 (0.21) | 2.40 (0.121) |
| rs12446632 | GPRC5B | 16:19842890 | A | G | -0.33 (0.21) | 2.42 (0.120) |
| rs12463617 | TMEM18 | 2:629244 | A | C | -0.54 (0.20) | 7.08 (0.008) |
| rs12517906 | NR | 5:180743819 | T | C | -0.04 (0.18) | 0.06 (0.808) |
| rs12566985 | FPGT-TNNI3K | 1:74774781 | G | A | 0.11 (0.15) | 0.50 (0.478) |
| rs12597579 | GP2 | 16:20246545 | T | C | 0.02 (0.30) | 0.01 (0.940) |
| rs12693973 | GALNT13 | 2:154314627 | G | T | 0.13 (0.17) | 0.58 (0.446) |
| rs12885454 | PRKD1 | 14:28806589 | A | C | -0.22 (0.16) | 1.83 (0.176) |
| rs12940622 | RPTOR | 17:76230166 | A | G | -0.02 (0.15) | 0.03 (0.874) |
| rs12964056 | MC4R | 18:60006567 | A | G | 0.30 (0.16) | 3.35 (0.067) |
| rs12970134 | MC4R | 18:60217517 | A | G | 0.34 (0.16) | 4.15 (0.042) |
| rs13021737 | TMEM18 | 2:622348 | A | G | -0.52 (0.20) | 6.49 (0.011) |
| rs13034723 | KLF9 | 2:190120954 | A | G | -0.09 (0.15) | 0.35 (0.554) |
| rs13078807 | CADM2 | 3:85835000 | G | A | 0.06 (0.18) | 0.11 (0.736) |
| rs13078960 | CADM2 | 3:85890280 | G | T | 0.07 (0.18) | 0.15 (0.702) |
| rs13107325 | SLC39A8 | 4:103407732 | T | C | 0.24 (0.29) | 0.70 (0.404) |
| rs13130484 | GNPDA2 | 4:45173674 | T | C | 0.20 (0.15) | 1.66 (0.198) |
| rs13191362 | PARK2 | 6:162953340 | G | A | 0.15 (0.22) | 0.49 (0.484) |
| rs13201877 | IFNGR1 | 6:137717234 | G | A | -0.04 (0.22) | 0.03 (0.857) |

| | | | | | | | | |
|---|---|---|---|---|---|---|---|---|
| rs1321847 | ELOVL4 | 6:79823470 | A | G | 0.16 | (0.24) | 0.45 | (0.503) |
| rs1333026 | intergenic | 13:65546652 | A | G | -0.64 | (0.23) | 8.07 | (0.005) |
| rs1406503 | ZNF804B | 7:88931564 | C | G | -0.44 | (0.43) | 1.03 | (0.310) |
| rs1440072 | KCNE4 | 2:223072020 | C | T | -0.09 | (0.30) | 0.10 | (0.755) |
| rs1441264 | MIR548A2 | 13:78478920 | G | A | -0.05 | (0.15) | 0.09 | (0.763) |
| rs1458095 | NR | 11:81274505 | T | C | -0.45 | (0.32) | 2.06 | (0.151) |
| rs1460676 | FIGN | 2:164275935 | C | T | 0.43 | (0.22) | 3.88 | (0.049) |
| rs1514175 | TNNI3K | 1:74525960 | A | G | 0.12 | (0.15) | 0.61 | (0.435) |
| rs1516725 | ETV5 | 3:187306698 | T | C | -0.12 | (0.23) | 0.27 | (0.602) |
| rs1528435 | UBE2E3 | 2:181259207 | C | T | -0.19 | (0.15) | 1.50 | (0.221) |
| rs1542829 | COL6A5 | 3:130418627 | A | G | -0.05 | (0.30) | 0.03 | (0.863) |
| rs1555543 | PTBP2 | 1:96479241 | A | C | 0.10 | (0.15) | 0.43 | (0.511) |
| rs1558902 | FTO | 16:52361075 | A | T | 0.40 | (0.15) | 7.13 | (0.008) |
| rs1561288 | POMC | 2:25146133 | T | C | -0.16 | (0.19) | 0.71 | (0.400) |
| rs16851483 | RASA2 | 3:142758126 | T | G | -0.32 | (0.38) | 0.69 | (0.405) |
| rs16858082 | GNPDA2 | 4:45173787 | C | T | 0.00 | (0.15) | 0.00 | (0.981) |
| rs16907751 | ZBTB10 | 8:81538012 | T | C | -0.12 | (0.28) | 0.18 | (0.671) |
| rs16951275 | MAP2K5 | 15:65864222 | C | T | -0.24 | (0.18) | 1.74 | (0.187) |
| rs17001654 | SCARB2 | 4:77348592 | G | C | 0.44 | (0.19) | 5.50 | (0.019) |
| rs17024393 | GNAT2 | 1:109956211 | C | T | 0.11 | (0.58) | 0.03 | (0.855) |
| rs17094222 | HIF1AN | 10:102385430 | C | T | -0.04 | (0.18) | 0.06 | (0.804) |
| rs17124318 | ATG4C | 1:63015059 | G | C | 0.00 | (0.42) | 0.00 | (0.991) |
| rs17203016 | CREB1 | 2:207963763 | G | A | -0.05 | (0.20) | 0.05 | (0.819) |
| rs17405819 | HNF4G | 8:76969139 | C | T | 0.13 | (0.17) | 0.58 | (0.448) |
| rs17724992 | PGPEP1 | 19:18315825 | G | A | -0.16 | (0.17) | 0.91 | (0.341) |
| rs17782313 | MC4R | 18:60183864 | C | T | 0.24 | (0.17) | 2.06 | (0.151) |
| rs17817964 | FTO | 16:53794154 | T | C | 0.45 | (0.15) | 9.04 | (0.003) |
| rs1808579 | C18orf8 | 18:19358886 | T | C | 0.10 | (0.15) | 0.42 | (0.516) |
| rs1878047 | NR | 19:51270548 | G | A | 0.04 | (0.16) | 0.08 | (0.778) |
| rs1927702 | NR | 9:15986718 | C | T | -0.14 | (0.16) | 0.84 | (0.360) |
| rs1928295 | TLR4 | 9:119418304 | C | T | -0.06 | (0.15) | 0.16 | (0.690) |
| rs2016586 | APOL5 | 22:35729217 | T | G | -0.15 | (0.16) | 0.86 | (0.353) |
| rs2030323 | BDNF | 11:27706992 | A | C | 0.06 | (0.18) | 0.10 | (0.756) |
| rs2033195 | GALNT10 | 5:154130036 | C | T | 0.11 | (0.16) | 0.50 | (0.480) |
| rs2033529 | TDRG1 | 6:40456631 | G | A | 0.16 | (0.17) | 0.96 | (0.328) |
| rs2033732 | RALYL | 8:85242264 | T | C | 0.14 | (0.18) | 0.64 | (0.425) |
| rs205262 | C6orf106 | 6:34671142 | G | A | -0.04 | (0.17) | 0.06 | (0.803) |
| rs206936 | HMGA1 | 6:34335092 | G | A | 0.15 | (0.19) | 0.60 | (0.437) |
| rs2075650 | TOMM40 | 19:50087459 | G | A | -0.17 | (0.26) | 0.45 | (0.504) |
| rs2080454 | CBLN1 | 16:47620091 | C | A | 0.04 | (0.16) | 0.06 | (0.813) |
| rs2112347 | POC5 | 5:75050998 | G | T | 0.21 | (0.16) | 1.73 | (0.188) |
| rs2121279 | LRP1B | 2:142759755 | T | C | 0.40 | (0.26) | 2.36 | (0.124) |
| rs2145270 | BMP2 | 20:6641038 | C | T | -0.18 | (0.16) | 1.26 | (0.262) |
| rs2176040 | LOC646736 | 2:226801046 | A | G | -0.10 | (0.16) | 0.40 | (0.528) |
| rs2176598 | HSD17B12 | 11:43820854 | T | C | 0.07 | (0.17) | 0.18 | (0.673) |
| rs2206734 | CDKAL1 | 6:20694653 | T | C | -0.08 | (0.21) | 0.15 | (0.697) |
| rs2207139 | TFAP2B | 6:50953449 | G | A | 0.51 | (0.20) | 6.69 | (0.010) |
| rs2236835 | PAX7 | 1:18663757 | A | G | -0.18 | (0.40) | 0.21 | (0.649) |
| rs2237892 | KCNQ1 | 11:2818521 | T | C | -0.16 | (0.35) | 0.20 | (0.655) |
| rs2241423 | LBXCOR1 | 15:67794500 | A | G | -0.22 | (0.18) | 1.45 | (0.229) |
| rs2275215 | LAMA2 | 6:129540247 | C | T | 0.24 | (0.16) | 2.19 | (0.139) |
| rs2287019 | QPCTL | 19:50894012 | T | C | -0.24 | (0.22) | 1.26 | (0.263) |
| rs2331841 | MC4R | 18:60161404 | A | G | 0.12 | (0.15) | 0.69 | (0.406) |
| rs2365389 | FHIT | 3:61211502 | T | C | -0.16 | (0.15) | 1.12 | (0.290) |
| rs2373011 | ANKS1B | 12:99567571 | G | C | 0.07 | (0.15) | 0.23 | (0.634) |
| rs2383393 | NR | 4:179743500 | A | G | -0.21 | (0.16) | 1.82 | (0.177) |
| rs2444217 | ADCY9 | 16:3988386 | G | A | -0.07 | (0.16) | 0.19 | (0.661) |
| rs2531995 | ADCY9 | 16:3963466 | C | T | 0.03 | (0.16) | 0.03 | (0.873) |
| rs2535633 | ITIH4 | 3:52825614 | G | C | -0.04 | (0.15) | 0.06 | (0.808) |
| rs255414 | HTR1A | 5:63522073 | G | A | 0.04 | (0.20) | 0.04 | (0.833) |
| rs2568958 | NEGR1 | 1:72299433 | G | A | -0.03 | (0.16) | 0.03 | (0.870) |
| rs2607292 | MARCH6 | 5:10372506 | T | C | -0.16 | (0.25) | 0.44 | (0.506) |
| rs261967 | PCSK1 | 5:96514546 | C | A | -0.03 | (0.16) | 0.03 | (0.866) |
| rs2650492 | SBK1 | 16:28240912 | A | G | -0.17 | (0.17) | 1.00 | (0.318) |
| rs2815752 | NEGR1 | 1:72346757 | G | A | -0.03 | (0.16) | 0.03 | (0.854) |
| rs2820292 | NAV1 | 1:200050910 | A | C | -0.12 | (0.15) | 0.66 | (0.415) |
| rs2836754 | ETS2 | 21:39213610 | T | C | -0.09 | (0.16) | 0.36 | (0.551) |
| rs2867125 | TMEM18 | 2:622827 | T | C | -0.54 | (0.20) | 6.91 | (0.009) |
| rs2890652 | LRP1B | 2:142202362 | C | T | 0.22 | (0.22) | 1.02 | (0.312) |
| rs2903492 | TMEM18 | 2:624678 | G | A | -0.56 | (0.20) | 7.46 | (0.006) |
| rs2922763 | HNF4G | 8:75661476 | G | T | 0.11 | (0.18) | 0.42 | (0.519) |
| rs2967951 | ROPN1L | 5:10463995 | T | C | -0.07 | (0.22) | 0.09 | (0.760) |

| | | | | | | | | |
|---|---|---|---|---|---|---|---|---|
| rs29941 | KCTD15 | 19:39001372 | A | G | 0.32 | (0.16) | 4.20 | (0.040) |
| rs3096490 | COL25A1 | 4:109057975 | A | G | 0.18 | (0.15) | 1.33 | (0.250) |
| rs3101336 | NEGR1 | 1:72523773 | T | C | -0.03 | (0.16) | 0.03 | (0.870) |
| rs348495 | GNPDA2 | 4:45182425 | A | G | 0.00 | (0.15) | 0.00 | (0.981) |
| rs3736485 | DMXL2 | 15:49535902 | A | G | 0.09 | (0.15) | 0.32 | (0.570) |
| rs3764400 | CBX1 | 17:48046570 | C | T | 0.28 | (0.21) | 1.69 | (0.194) |
| rs3803915 | NR | 19:2160530 | A | C | -0.46 | (0.26) | 3.09 | (0.079) |
| rs3810291 | ZC3H4 | 19:52260843 | G | A | 0.07 | (0.17) | 0.18 | (0.672) |
| rs3817334 | MTCH2 | 11:47607569 | T | C | 0.06 | (0.15) | 0.18 | (0.668) |
| rs3849570 | GBE1 | 3:81874802 | A | C | -0.06 | (0.16) | 0.14 | (0.704) |
| rs3888190 | ATP2A1 | 16:28796987 | A | C | -0.04 | (0.16) | 0.06 | (0.813) |
| rs3934834 | NR | 1:1070426 | T | C | 0.12 | (0.22) | 0.31 | (0.575) |
| rs3936060 | ZNF608 | 5:125011395 | G | C | -0.08 | (0.19) | 0.16 | (0.689) |
| rs4256980 | TRIM66 | 11:8630515 | C | G | -0.09 | (0.16) | 0.36 | (0.551) |
| rs4377469 | CCK | 3:42261582 | G | T | -0.01 | (0.24) | 0.00 | (0.966) |
| rs4432245 | EIF2AK4 | 15:40032280 | C | T | 0.10 | (0.34) | 0.09 | (0.760) |
| rs4740619 | C9orf93 | 9:15624326 | C | T | 0.09 | (0.16) | 0.35 | (0.555) |
| rs4771122 | GTF3A | 13:27446043 | G | A | 0.07 | (0.16) | 0.17 | (0.680) |
| rs4776970 | MAP2K5 | 15:67788548 | T | A | -0.36 | (0.16) | 4.86 | (0.028) |
| rs4787491 | INO80E | 16:29922838 | A | G | 0.04 | (0.16) | 0.06 | (0.808) |
| rs4836133 | ZNF608 | 5:124996410 | C | A | -0.16 | (0.15) | 1.02 | (0.312) |
| rs4854307 | intergenic | 2:443281 | C | T | -0.14 | (0.35) | 0.16 | (0.692) |
| rs492400 | USP37 | 2:219057996 | C | T | 0.12 | (0.15) | 0.64 | (0.422) |
| rs4929949 | RPL27A | 11:8583046 | C | T | 0.02 | (0.15) | 0.03 | (0.868) |
| rs516636 | SEC16B | 1:177886382 | T | G | -0.11 | (0.19) | 0.34 | (0.558) |
| rs543874 | SEC16B | 1:176156103 | G | A | -0.09 | (0.19) | 0.21 | (0.644) |
| rs571312 | MC4R | 18:60172536 | A | C | 0.23 | (0.17) | 1.88 | (0.171) |
| rs574367 | SEC16B | 1:177904075 | T | G | -0.13 | (0.19) | 0.44 | (0.509) |
| rs591166 | MC4R | 18:60174356 | A | T | 0.15 | (0.15) | 0.97 | (0.324) |
| rs6091540 | ZFP64 | 20:50521269 | T | C | -0.19 | (0.17) | 1.23 | (0.267) |
| rs6107853 | BMP2 | 20:6629230 | A | G | -0.02 | (0.15) | 0.02 | (0.891) |
| rs62033400 | FTO | 16:53777876 | G | A | 0.46 | (0.15) | 9.27 | (0.002) |
| rs6265 | BDNF | 11:27658369 | T | C | 0.16 | (0.18) | 0.77 | (0.382) |
| rs633715 | SEC16B | 1:177883445 | C | T | -0.12 | (0.20) | 0.36 | (0.548) |
| rs6465468 | ASB4 | 7:95007450 | T | G | 0.02 | (0.16) | 0.02 | (0.884) |
| rs6477694 | EPB41L4B | 9:110972163 | C | T | 0.13 | (0.16) | 0.61 | (0.436) |
| rs6499640 | FTO | 16:53735765 | G | A | -0.54 | (0.16) | 11.19 | (0.001) |
| rs652722 | PAX6 | 11:31883988 | T | C | 0.18 | (0.18) | 0.93 | (0.334) |
| rs6545814 | ADCY3 | 2:24908447 | G | A | 0.00 | (0.15) | 0.00 | (0.995) |
| rs6548238 | TMEM18 | 2:634905 | T | C | -0.45 | (0.20) | 4.86 | (0.027) |
| rs6567160 | MC4R | 18:55980115 | C | T | 0.22 | (0.17) | 1.78 | (0.183) |
| rs657452 | AGBL4 | 1:49362434 | A | G | 0.40 | (0.15) | 7.11 | (0.008) |
| rs6794092 | PP13439 | 3:171840554 | A | G | 0.89 | (0.58) | 2.33 | (0.127) |
| rs6804842 | RARB | 3:25081441 | A | G | 0.06 | (0.15) | 0.14 | (0.708) |
| rs6893807 | LINC00461 | 5:88669203 | G | A | -0.34 | (0.20) | 2.83 | (0.093) |
| rs713586 | ADCY3 | 2:24935139 | C | T | 0.01 | (0.15) | 0.01 | (0.921) |
| rs7138803 | BCDIN3D | 12:48533735 | A | G | -0.03 | (0.16) | 0.03 | (0.866) |
| rs7141420 | NRXN3 | 14:78969207 | C | T | -0.14 | (0.15) | 0.89 | (0.344) |
| rs7164727 | LOC100287559 | 15:70881044 | C | T | -0.18 | (0.17) | 1.13 | (0.288) |
| rs7202116 | FTO | 16:53787703 | G | A | 0.47 | (0.15) | 9.53 | (0.002) |
| rs7234864 | MC4R | 18:60067625 | T | C | 0.16 | (0.17) | 0.88 | (0.348) |
| rs7239883 | LOC284260 | 18:38401669 | G | A | -0.10 | (0.15) | 0.42 | (0.519) |
| rs7243357 | GRP | 18:55034299 | G | T | -0.11 | (0.21) | 0.28 | (0.600) |
| rs7359397 | AC138894.2 | 16:28874338 | T | C | -0.03 | (0.16) | 0.03 | (0.854) |
| rs7481311 | BDNF | 11:27561582 | T | C | 0.35 | (0.18) | 3.49 | (0.062) |
| rs7498665 | SH2B1 | 16:28871920 | G | A | -0.04 | (0.16) | 0.06 | (0.813) |
| rs7561317 | TMEM18 | 2:644953 | A | G | -0.43 | (0.20) | 4.44 | (0.035) |
| rs7586879 | ADCY3 | 2:24894108 | T | C | 0.10 | (0.16) | 0.39 | (0.535) |
| rs758747 | NLRC3 | 16:3567359 | T | C | 0.18 | (0.16) | 1.23 | (0.267) |
| rs7599312 | ERBB4 | 2:213121476 | A | G | -0.33 | (0.18) | 3.40 | (0.065) |
| rs7647305 | DGKG | 3:186116501 | T | C | -0.09 | (0.18) | 0.22 | (0.639) |
| rs7708584 | GALNT10 | 5:154163906 | A | G | 0.10 | (0.16) | 0.41 | (0.524) |
| rs7715256 | GALNT10 | 5:153518086 | G | T | 0.10 | (0.16) | 0.41 | (0.524) |
| rs7899106 | GRID1 | 10:87400884 | G | A | 0.07 | (0.34) | 0.04 | (0.840) |
| rs7903146 | TCF7L2 | 10:114748339 | T | C | -0.20 | (0.17) | 1.49 | (0.222) |
| rs8005845 | SERPINA3 | 14:94628060 | C | T | 0.19 | (0.17) | 1.29 | (0.257) |
| rs8050136 | FTO | 16:53782363 | A | C | 0.46 | (0.15) | 9.26 | (0.002) |
| rs8089364 | MC4R | 18:60191596 | C | T | 0.34 | (0.16) | 4.24 | (0.039) |
| rs817858 | RAD23B | 9:107363530 | G | C | -1.03 | (0.62) | 2.77 | (0.096) |
| rs8192472 | CCK | 3:42258378 | T | C | 0.06 | (0.16) | 0.13 | (0.715) |
| rs824931 | NR | 2:221936980 | C | T | 0.26 | (0.16) | 2.85 | (0.091) |

| | | | | | | |
|---|---|---|---|---|---|---|
| rs867559 | LMX1B | 9:126703046 | G | A | -0.14 (0.21) | 0.50 (0.482) |
| rs887912 | FANCL | 2:59075742 | T | C | 0.26 (0.17) | 2.22 (0.136) |
| rs888789 | FLJ35779 | 5:75692544 | G | A | 0.11 (0.16) | 0.46 (0.496) |
| rs925946 | BDNF | 11:27645655 | T | G | 0.34 (0.17) | 4.12 (0.042) |
| rs933117 | GRIK1 | 21:29728478 | A | G | -0.10 (0.31) | 0.11 (0.741) |
| rs9356744 | CDKAL1 | 6:20685255 | C | T | 0.12 (0.17) | 0.52 (0.469) |
| rs9374842 | LOC285762 | 6:120227364 | C | T | -0.22 (0.18) | 1.49 (0.222) |
| rs939583 | TMEM18 | 2:622531 | C | T | -0.56 (0.20) | 7.46 (0.006) |
| rs9400239 | FOXO3 | 6:109084356 | T | C | 0.02 (0.16) | 0.01 (0.906) |
| rs9473924 | TFAP2B | 6:50866444 | T | G | 0.31 (0.17) | 3.43 (0.064) |
| rs9540493 | MIR548X2 | 13:65103705 | A | G | -0.13 (0.15) | 0.72 (0.396) |
| rs955423 | TENM2 | 5:166292645 | C | A | -0.08 (0.15) | 0.25 (0.618) |
| rs9568867 | OLFM4 | 13:53533217 | A | G | -0.02 (0.22) | 0.01 (0.931) |
| rs9641123 | CALCR | 7:93035668 | C | G | -0.24 (0.16) | 2.28 (0.131) |
| rs974417 | KLHL32 | 6:96971722 | T | C | -0.02 (0.22) | 0.01 (0.916) |
| rs977747 | TAL1 | 1:47457264 | T | G | -0.18 (0.16) | 1.29 (0.256) |
| rs9816226 | ETV5 | 3:186116710 | A | T | -0.11 (0.20) | 0.30 (0.587) |
| rs987237 | TFAP2B | 6:50835337 | G | A | 0.38 (0.19) | 3.88 (0.049) |
| rs9906155 | NR | 17:77705615 | C | T | 0.41 (0.29) | 1.96 (0.162) |
| rs9914578 | SMG6 | 17:1951886 | G | C | 0.11 (0.20) | 0.34 (0.557) |
| rs9925964 | KAT8 | 16:31037396 | G | A | -0.03 (0.15) | 0.05 (0.817) |
| rs9939609 | FTO | 16:53786615 | A | T | 0.47 (0.15) | 9.53 (0.002) |
| rs9940128 | FTO | 16:53766842 | A | G | 0.38 (0.15) | 6.44 (0.011) |

SNPs are listed by NCBI's rs number. Grey-shaded SNPs correspond to SNPs having a significant association with obesity, defined as a binary variable (body mass index ≥ 30kg/m$^2$), in the Quebec Family Study (QFS) discovery sample. SNP: single nucleotide polymorphism. Reported gene: gene reported by authors in the NHGRI-EBI GWAS Catalog. Position: SNP localization (chromosome:base pair) in the reference genome GRCh38/hg38. $\chi^2$ corresponds to the association results with obesity. β (SE): beta coefficient of association and standard error.

**References**

1. Locke AE et al. Genetic studies of body mass index yield new insights for obesity biology. Nature 2015;518(7538):197–206.

2. Croteau-Chonka DC et al. Genome-wide association study of anthropometric traits and evidence of interactions with age and study year in Filipino women. Obesity (Silver Spring). 2011;19(5):1019–27.

3. Fox CS et al. Genome-wide association to body mass index and waist circumference: the Framingham Heart Study 100K project. BMC Med. Genet. 2007;8 Suppl 1(Suppl 1):S18.

4. Graff M et al. Genome-wide analysis of BMI in adolescents and young adults reveals additional insight into the effects of genetic loci over the life course. Hum. Mol. Genet. 2013;22(17):3597–607.

5. Johansson Å et al. Linkage and genome-wide association analysis of obesity-related phenotypes: Association of weight with the MGAT1 gene. Obesity 2010;18(4):803–808.

6. Liu JZ et al. Genome-wide association study of height and body mass index in Australian twin families. Twin Res. Hum. Genet. 2010;13(2):179–93.

7. Loos RJF et al. Common variants near MC4R are associated with fat mass, weight and risk of obesity. Nat. Genet. 2008;40(6):768–75.

8. Namjou B et al. EMR-linked GWAS study: investigation of variation landscape of loci for body mass index in children. Front. Genet. 2013;4:268.

9. Ng MCY et al. Genome-wide association of BMI in African Americans. Obesity 2012;20(3):622–627.

10. Okada Y et al. Common variants at CDKAL1 and KLF9 are associated with body mass index in east Asian populations. Nat. Genet. 2012;44(3):302–6.

11. Scannell Bryan M et al. Genome-wide association studies and heritability estimates of body mass index related phenotypes in Bangladeshi adults. PLoS One 2014;9(8):e105062.

12. Speliotes EK et al. Association analyses of 249,796 individuals reveal 18 new loci associated with body mass index. Nat. Genet. 2010;42(11):937–948.

13. Thorleifsson G et al. Genome-wide association yields new sequence variants at seven loci that associate with measures of obesity. Nat. Genet. 2009;41(1):18–24.

14. Willer CJ et al. Six new loci associated with body mass index highlight a neuronal influence on body weight regulation. Nat. Genet. 2009;41(1):25–34.

15. Yang F et al. Genome wide association study: searching for genes underlying body mass index in the Chinese. Biomed. Environ. Sci. 2014;27(5):360–70.

16. Yang J et al. FTO genotype is associated with phenotypic variability of body mass index. Nature 2012;490(7419):267–72.

17. Berndt SI et al. Genome-wide meta-analysis identifies 11 new loci for anthropometric traits and provides insights into genetic architecture. Nat. Genet. 2013;45(5):501–12.

18. Monda KL et al. A meta-analysis identifies new loci associated with body mass index in individuals of African ancestry. Nat. Genet. 2013;45(6):690–6.

19. Pei Y-F et al. Meta-analysis of genome-wide association data identifies novel susceptibility loci for obesity. Hum. Mol. Genet. 2014;23(3):820–30.

20. Wang KS et al. A novel locus for body mass index on 5p15.2: A meta-analysis of two genome-wide association studies. Gene 2012;500(1):80–84.

21. Wen W et al. Meta-analysis of genome-wide association studies in East Asian-ancestry populations identifies four new loci for body mass index. Hum. Mol. Genet. 2014;23(20):5492–504.

22. Wen W et al. Meta-analysis identifies common variants associated with body mass index in east Asians. Nat. Genet. 2012;44(3):307–11.
